# Sodium Humate-Derived Gut Microbiota Ameliorates Intestinal Dysfunction Induced by *Salmonella* Typhimurium in Mice

**DOI:** 10.1128/spectrum.05348-22

**Published:** 2023-04-17

**Authors:** Dong Wang, Yingce Zheng, Yuying Fan, Yanjun He, Kexin Liu, Shouxiang Deng, Yun Liu

**Affiliations:** a Heilongjiang Key Laboratory of Experimental Animals and Comparative Medicine, College of Veterinary Medicine, Northeast Agricultural University, Harbin, China; b College of Veterinary Medicine, Shandong Agricultural University, Tai’an, China; University of California Davis

**Keywords:** *Salmonella* Typhimurium, sodium humate, gut microbiota, fecal microbiota transplantation, intestinal barrier function

## Abstract

*Salmonella* is a foodborne pathogen that is one of the main causes of gastroenteric disease in humans and animals. As a natural organic substance, sodium humate (HNa) possesses antibacterial, antidiarrheal, and anti-inflammatory properties. However, it is unclear whether the HNa and HNa-derived microbiota exert alleviative effects on Salmonella enterica serovar Typhimurium-induced enteritis. We found that treatment with HNa disrupted the cell wall of *S*. Typhimurium and decreased the virulence gene expression. Next, we explored the effect of HNa presupplementation on *S*. Typhimurium-induced murine enteritis. The results revealed that HNa ameliorated intestinal pathological damage. In addition, we observed that presupplementation with HNa enhanced intestinal barrier function via modulating gut microbiota, downregulating toll-like receptor 4 (TLR4)/nuclear factor kappa-B (NF-κB) and NOD-like receptor protein 3 (NLRP3) signaling pathways, regulating intestinal mucosal immunity, and enhancing tight junction protein expression. To further validate the effect of HNa-derived microbiota on *S*. Typhimurium-induced enteritis, we performed fecal microbiota transplantation and found that HNa-derived microbiota also alleviated *S*. Typhimurium-induced intestinal damage. It is noteworthy that both HNa and HNa-derived microbiota improved the liver injury caused by *S*. Typhimurium infection. Collectively, this is the first study to confirm that HNa could alleviate *S*. Typhimurium-induced enteritis in a gut microbiota-dependent manner. This study provides a new perspective on HNa as a potential drug to prevent and treat salmonellosis.

**IMPORTANCE** *Salmonella* Typhimurium is an important zoonotic pathogen, widely distributed in nature. *S*. Typhimurium is one of the leading causes of foodborne illnesses worldwide, and more than 350,000 people died from *Salmonella* infection each year, which poses a substantial risk to public health and causes a considerable economic loss. Here, we found that the *S*. Typhimurium infection caused severe intestinal and liver damage. In addition, we first found that sodium humate (HNa) and HNa-derived gut microbiota can alleviate *S*. Typhimurium infection-induced intestinal damage. These findings extend the knowledge about the public health risk and pathogenic mechanisms of *S*. Typhimurium.

## INTRODUCTION

*Salmonella*, one of the most prevalent foodborne zoonoses worldwide, can cause severe intestinal injury by damaging the epithelium and disturbing the gut microflora homeostasis (1). It has been estimated that more than 350,000 people die from *Salmonella* infection worldwide each year, which poses a substantial risk to public health and causes considerable economic loss (2). The intestinal barrier is the first line of defense against invasive pathogens and potentially harmful substances (3). Therefore, the intact intestinal barrier and stable gut microecology are of great importance against pathogenic microorganisms and maintaining intestinal homeostasis (4). Studies have shown that *Salmonella* adhering to intestinal epithelial cells (IECs) could weaken the expression of cell tight junctions and adherence junctions, increase intestinal permeability, and accelerate intestinal inflammatory responses and oxidative stress, ultimately resulting in intestinal barrier dysfunction (5). At present, antibiotic therapy is the main treatment for salmonellosis, while a worldwide concern has been raised over the emergence of antibiotic resistance and adverse public health outcomes (6). Furthermore, long-term use of antibiotics could disrupt the composition of gut microbiota (7). More seriously, as an intracellular pathogen, Salmonella enterica serovar Typhimurium can infect and survive both phagocytic and nonphagocytic cells (8). These properties further reduce the effectiveness of antibiotics against *S*. Typhimurium. Taken together, natural products with antibacterial and anti-inflammatory activities as well as regulating effects on gut homeostasis have attracted more attention for the prevention and treatment of salmonellosis.

Humic acids (HAs) are formed by decomposing and transforming organic matter in the peat and are widely applied in multiple fields, such as agriculture, medicine and health, and environmental protection (9). Sodium humate (HNa), a salt of HAs, has been widely used as a therapeutic agent in traditional Chinese medicine for hemostasis and antidiarrhea with antimicrobial, anti-inflammatory, immunoregulatory, antirheumatic, and wound healing repairing properties (10). Our previous study demonstrated that HNa strongly inhibited the proliferation of Escherichia coli *in vitro* and *in vivo* and further alleviated intestinal damage induced by E. coli through modulating gut microflora and suppressing intestinal inflammatory responses (11). Similarly, HNa supplements reduced E. coli colonization in the gut of piglets with enterotoxigenic Escherichia coli (ETEC) infection according to a previous study (12). In addition, the immunoregulatory and anti-inflammatory properties of HNa have been extensively reported in piglets, mice, broilers, and European *seabass* (13–15). Interestingly, it was indicated that HA can effectively alleviate polystyrene nanoplastic particle toxicity to Daphnia magna (16). These results support our hypothesis that HNa may exert ameliorative effects on intestinal injury induced by *S*. Typhimurium infection. Nevertheless, the mechanism by which the action takes place remains unclear.

Gut microbiota, one of the important constituents of the intestinal mucosal barrier, is essential for sustaining intestinal barrier function (17). The occurrence and development of various diseases are closely linked to gut microflora disturbance (18). Extensive studies have shown that *Salmonella* infection can disrupt gut microflora homeostasis (19, 20). Recent research elucidated that fecal microbiota transplantation (FMT) can treat intestinal diseases by regulating gut microbiota (21). Microbiological diversity and composition in the feces are crucial for FMT efficiency. Correspondingly, HNa can regulate gut microbiota by increasing the abundance of gut beneficial bacteria (11). In the present study, we firstly determined the optimal protective concentration of HNa against *S*. Typhimurium and then investigated the alleviative effects of HNa presupplementation on intestinal damage in *S*. Typhimurium-infected mice through a systematic evaluation of intestinal inflammatory response, intestinal mucosal immunity, gut microbiota, and intestinal barrier function. Finally, we demonstrated that HNa-derived gut microbiota could alleviate intestinal damage in *S*. Typhimurium-infected mice.

## RESULTS

### *S.* Typhimurium morphology and virulence gene expression.

Scanning electron microscopy (SEM) and reverse transcription-quantitative PCR (qRT-PCR) were used to investigate the effect of HNa on *S*. Typhimurium morphology and virulence gene expression. The results showed that untreated *S*. Typhimurium exhibited a regular cell shape with intact cell membranes. By contrast, *S*. Typhimurium treated with HNa revealed severe damage of the cell structure and further cell wall shrinkage, damage, and lysis (see Fig. S1A in the supplemental material). Next, we further evaluated gene expression associated with *S*. Typhimurium motility (*flgG* and *motA*), adhesion and invasion (*sopB*, *invh*, *sipa*, and *sipb*), and cell wall integrity (*hflk*, *lrp*, *ompr*, and *tata*). The results showed that treatment with HNa significantly decreased the gene expression of *flgG*, *sopB*, *invh*, *sipa*, *hflk*, and *ompr* compared with that of the untreated *S*. Typhimurium (*P *< 0.05) (see Fig. S1B to D).

To further explore the effect of HNa on resident gut microflora and *S*. Typhimurium *in vivo*, we used SS agar plates and MacConkey plates to isolate E. coli and *S*. Typhimurium in the feces of HNa pretreatment mice. The results of SEM found no significant effect of HNa on the morphology of *S*. Typhimurium and E. coli *in vivo* (Fig. S1A). In summary, these results suggest that HNa disrupts the cell morphology and reduces the gene expression of virulence factors in *S*. Typhimurium *in vitro* but not *in vivo*.

### HNa alleviated *S*. Typhimurium-induced intestinal damage and oxidative stress.

**FIG 1:**
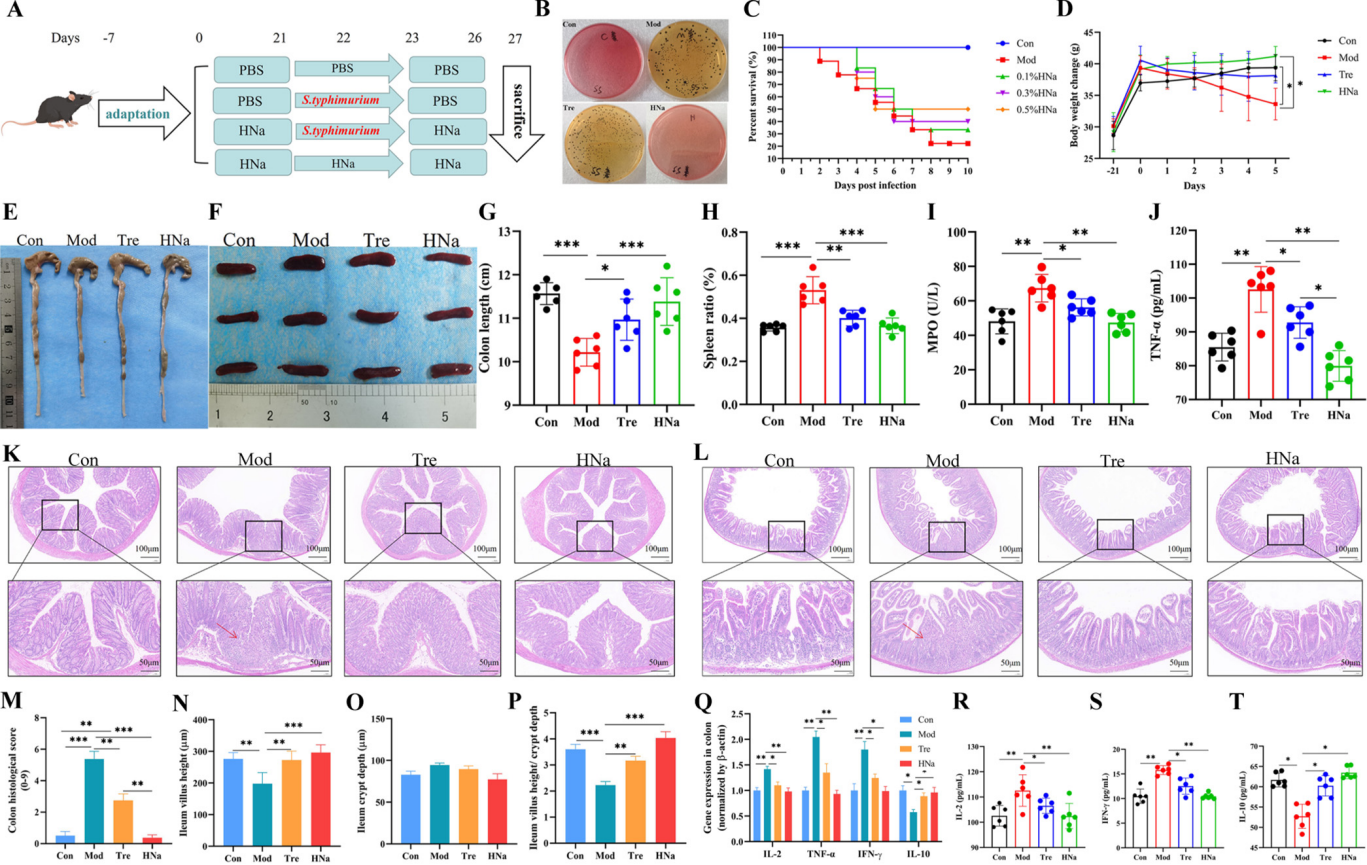
Administration of HNa alleviated *Salmonella* Typhimurium-induced intestinal damage. (A) Animal experimental design. (B) The colonization of *S*. Typhimurium was tested. (C) The optimal protective concentration of HNa against *S*. Typhimurium-infected mice. (D) Daily body weight changes throughout the duration of the study. (E, G) The lengths of the colon. (F) Macroscopic pictures of the spleen. (H) Spleen weight/body weight ratio. (I) The activity of serum MPO. The concentrations of tumor necrosis factor-α (TNF-α) (J), interleukin-2 (IL-2) (R), interferon-γ (IFN-γ) (S), and interleukin-10 (IL-10) (T) in the serum and the mRNA expression (Q) in the colon. H&E-stained colon sections (K) and histological scores of colons (M). H&E-stained ileum sections (L) and villus height (N), crypt depth (O), and the ratio of villus height and crypt depth of ileum (P). Data were presented as means ± SD. Statistical significance was determined using one-way ANOVA, followed by Tukey test. *, *P* < 0.05; **, *P* < 0.01; ***, *P* < 0.001.

**FIG 2:**
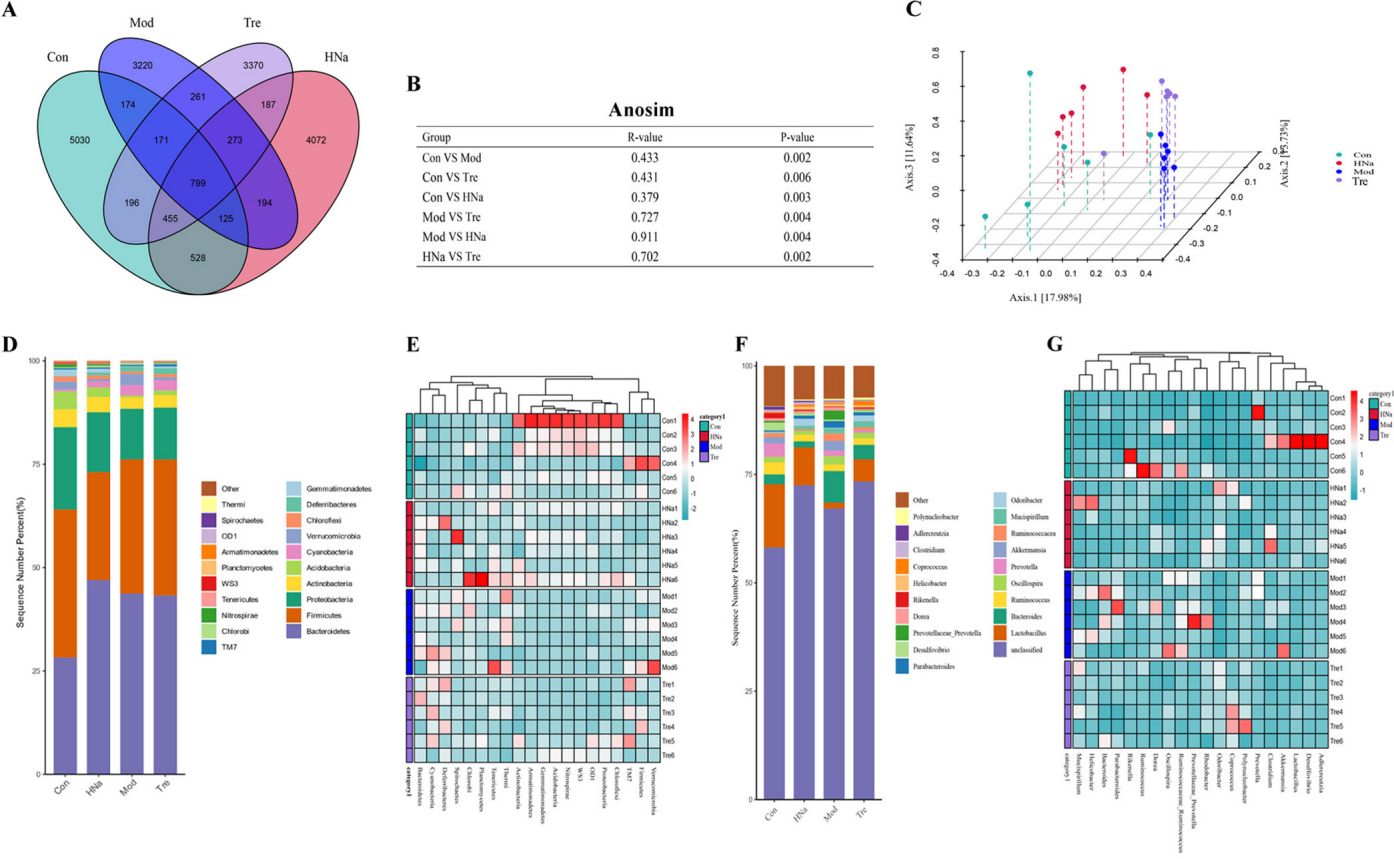
Administration of sodium humate (HNa) regulated the composition of gut microbiota. (A) A Venn diagram displayed the overlaps among groups. (B) ANOSIM analysis among groups (*n* = 10). (C) Principal coordinates analysis (PCoA) plot of gut microbiota, each represented by one color (*n* = 10). Community bar plots and heatmap of phylum (D, E) and genus (F, G).

To investigate whether *S*. Typhimurium can colonize the intestinal tract and optimum HNa protective concentration, mouse feces were collected after *S*. Typhimurium infection. The results showed that *S*. Typhimurium successfully colonized the intestinal tract, and the optimal protective concentration of HNa was 0.5% (Fig. 1B and C). In addition, as shown in Fig. 2, compared with the Mod group, HNa alleviated *S*. Typhimurium-induced intestinal damage, as evidenced by the decreased body weight loss and spleen index (Fig. 1D and H), reduced colon shortening and histological score (Fig. 1G and M), elevated ileum villus height (Fig. 2N), and decreased blood counts of leucocytes, lymphocyte, and granulocytes (see Fig. S2A in the supplemental material). The results of hematoxylin-eosin (H&E) staining (Fig. 1K and L) revealed destruction of the intestinal barrier and mucosal edema in the ileum and colon of the *S*. Typhimurium-infected mice, while HNa pretreatment reversed that.

To further assess the effects of HNa on inflammatory responses, the activity of serum myeloperoxidase (MPO), and the levels of serum and colon inflammatory cytokines were measured. The results found that *S*. Typhimurium potently elevated the activity of serum MPO compared with Con, HNa, and Tre groups (*P* < 0.05) (Fig. 1I). Similarly, serum concentrations of necrosis factor alpha (TNF-α) (Fig. 1J), interleukin-2 (IL-2) (Fig. 1R), and gamma interferon (IFN-γ) (Fig. 2S) were significantly increased, while IL-10 (Fig. 1T) was significantly decreased in *S*. Typhimurium-infected mice, and the mRNA expression levels of IL-2, IFN-γ, TNF-α, and IL-10 in colonic tissue are consistent with those in the serum (Fig. 1Q). Above all, these changes were positively improved by HNa pretreatment (*P* < 0.05).

To assess the effect of HNa on oxidative stress caused by *S*. Typhimurium, total superoxide dismutases (T-SOD), total antioxidant capacity (T-AOC), catalase (CAT), glutathione (GSH), and malondialdehyde (MDA) in the serum were measured. The results revealed that *S*. Typhimurium infection significantly decreased the activities of T-SOD, T-AOC, and GSH but increased the concentration of MDA in the serum of mice compared with Con and HNa groups (*P *< 0.05) (Fig. S2B to F). Interestingly, this phenomenon was largely attenuated by HNa pretreatment. To summarize, pretreatment with HNa alleviated *S*. Typhimurium-induced intestinal damage, decreased the levels of inflammatory cytokines, and improved resistance to oxidative stress.

### HNa regulated the composition of gut microbiota.

The impact of HNa on the gut microbiota composition of *S*. Typhimurium-infected mice was investigated. The Venn diagram (Fig. 2A) showed that there were 799 shared operational taxonomic unit (OTUs) among four groups, and 5,030, 3,220, 3,370, and 4,072 specific OTUs were observed in the Con, Mod, Tre, and HNa groups, respectively. Alpha diversity was similar among groups (Fig. S2G to K). The plots of principal coordinate analysis (PCoA) and analysis of similarity (ANOSIM) based on Bray-Curtis distance showed a separation in the gut microbiota structure among groups, which indicated that the gut microbiota structure was significantly influenced by *S*. Typhimurium or HNa (Fig. 2B and C).

**FIG 3:**
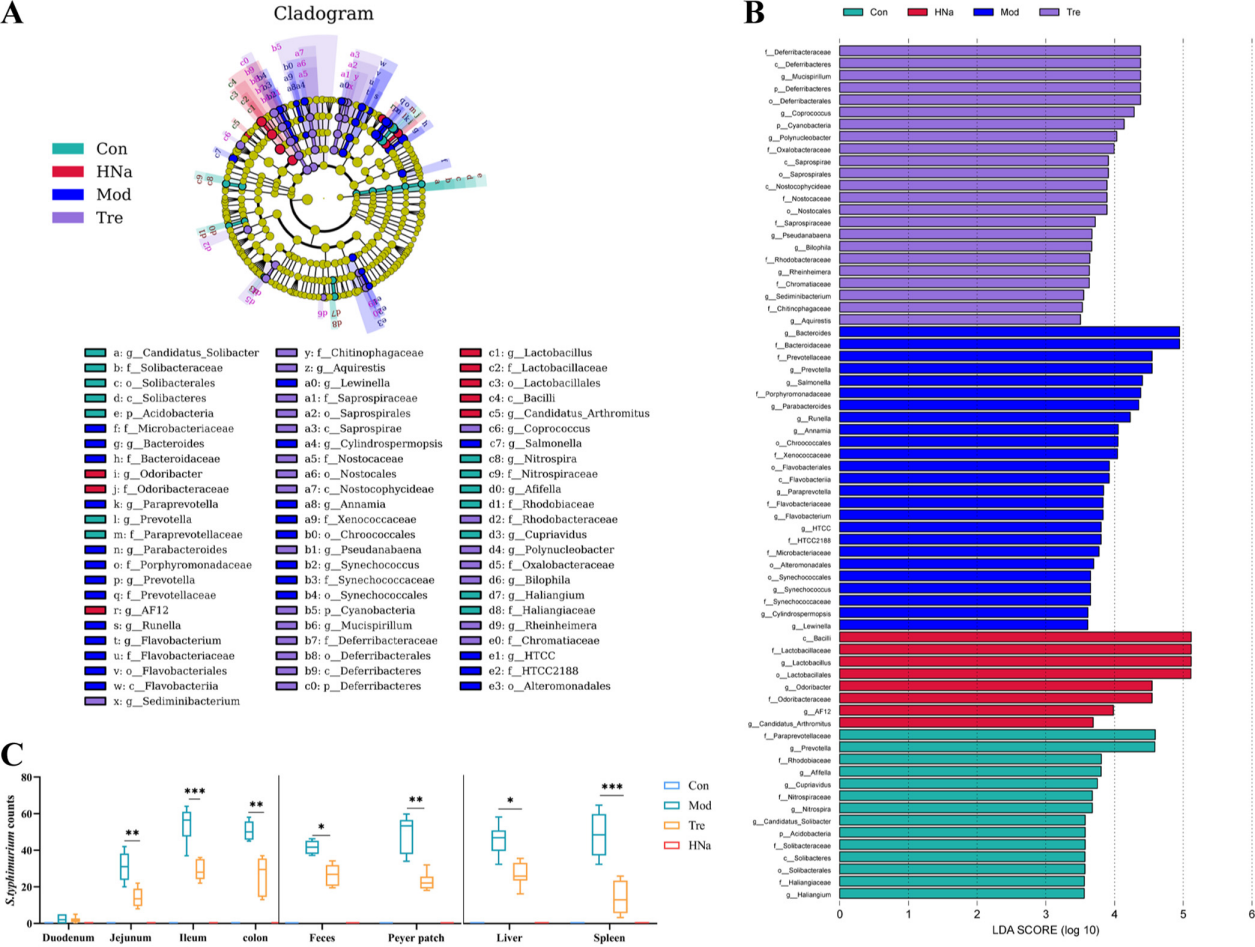
Administration of sodium humate (HNa) regulated the composition and function of gut microbiota. (A) Taxonomic cladogram of linear discriminant analysis effect size (LEfSe) analysis. Different colors indicate the enrichment of the biomarker taxa in different groups. The circle from inside to outside means the rank from kingdom to species, and the circle size represents the taxa abundance in the community. (B) Linear discriminant analysis (LDA) scores for different taxa abundances. (C) The number of *S*. Typhimurium in the duodenum, jejunal, ileal, colon and feces, payer patch, liver, and spleen (*n* = 10). Data were presented as means ± SD. Statistical significance was determined using one-way ANOVA, followed by Tukey test. *, *P* < 0.05; **, *P* < 0.01; ***, *P *< 0.001.

At the phylum level, the gut microbiota was dominated by *Firmicutes*, *Bacteroidetes*, and *Proteobacteria* (Fig. 2D). At the genus level, the gut microbiota was dominated by *Lactobacillus*, *Bacteroides*, and *Ruminococcus* (Fig. 2F). The linear discriminant analysis effect size (LEfSe) (Fig. 3A) and linear discriminant analysis (LDA) found that 12 bacterial genera, including *Salmonella*, *Prevotella*, and *Bacteroides* were enriched in the Mod group, while 8 bacterial genera including *Mucispirillum*, *Coprococcus*, and *Polynucleobacter* were enriched in the Tre group. In addition, 4 bacterial genera including *Lactobacillus* and *Odoribacter* were enriched in the HNa group (Fig. 3B).

As shown, *Salmonella* abundance in the feces was enriched in the Mod group based on LDA analysis. Thus, the numbers of *S*. Typhimurium cell in mice intestine, liver, payer patch, spleen, and feces were examined. As shown in Fig. 3C, compared with the Con and HNa groups, *S*. Typhimurium infection increased the numbers of *S*. Typhimurium cells in the jejunum, ileum, colon, feces, liver, payer patch, and spleen (*P *< 0.05). However, pretreatment with HNa significantly reduced the number of *S*. Typhimurium cells compared with the Mod group (*P *< 0.05), indicating the strong antimicrobial activity of HNa. Taken together, *S*. Typhimurium infection significantly influenced the gut microbiota structure, while pretreatment with HNa restored the balance of the gut microbiota and increased the abundance of beneficial bacteria, suggesting that HNa has an effect on modulating gut microbiota.

### HNa alleviated intestinal inflammation.

**FIG 4:**
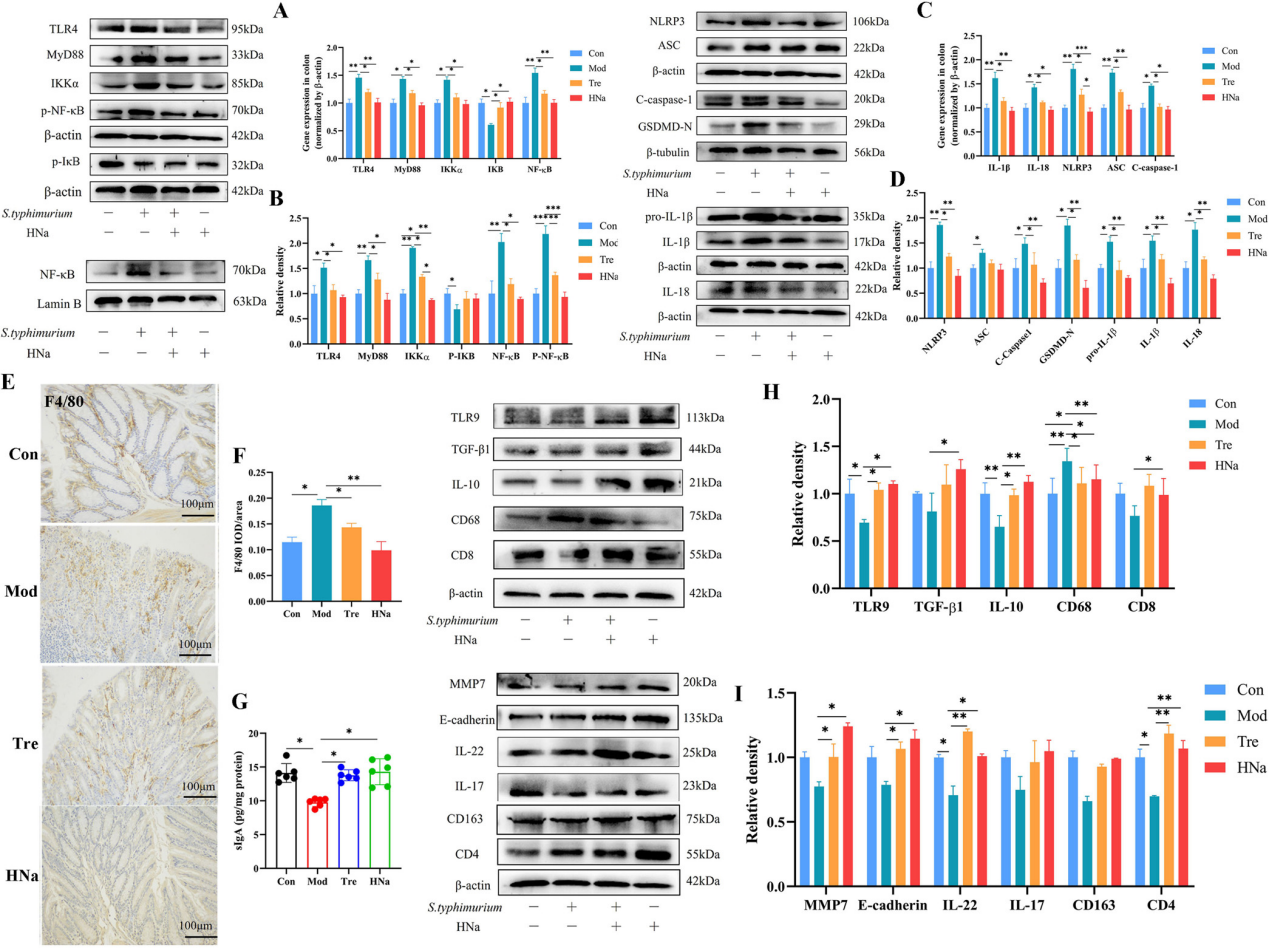
Administration of sodium humate (HNa) regulated the intestinal mucosa immunity and intestinal inflammation in *Salmonella* Typhimurium-infected mice. The mRNA (A) and protein (B) expression of TLR4/NF-κB signaling pathway-relevant proteins. The mRNA (C) and protein (D) expression of NOD-like receptor protein 3 (NLRP3) inflammasome-relevant proteins. Microscopic images of colonic tissues stained with immunohistochemical of F4/80 (E) and mean optical densities (F). (G) The concentration of secretory immunoglobulin A (sIgA) in the colon. (H) The protein expression of toll-like receptor 9 (TLR9), transforming growth factor-β1 (TGF-β1), interleukin-10 (IL-10), cluster of differentiation 68 (CD68), and cluster of differentiation 8 (CD8) in the colon. (I) The protein expression of matrix metalloproteinase 7 (MMP7), E-cadherin, interleukin-22 (IL-22), interleukin-17 (IL-17), cluster of differentiation 163 (CD163), and cluster of differentiation 4 (CD4) in the colon. Data were presented as means ± SD. Statistical significance was determined using one-way ANOVA, followed by Tukey test. *, *P* < 0.05; **, *P* < 0.01; ***, *P* < 0.001. Note that the results shown in panels A and C are from the same experiment, and therefore, the blots labeled “β-actin” are identical.

To investigate whether HNa pretreatment can alleviate intestinal inflammation caused by *S*. Typhimurium infection by regulating intestinal mucosal immunity, we determined the concentration of secretory immunoglobulin A (sIgA) in the colon. The results revealed that HNa pretreatment increased the concentration of sIgA in the colon when compared with that of the Mod group (*P *< 0.05) (Fig. 4G). Excessive macrophage infiltration promotes the development of chronic inflammation. In addition, in situations of tissue injury or infection, macrophages can switch between the proinflammatory M1 type and the anti-inflammatory M2 type; therefore, we investigated whether HNa would favor the M2 type. The results showed that protein expression of M1 macrophage markers F4/80 and CD68 was upregulated (Fig. 4F and H), while M2 macrophage marker IL-10 was downregulated in the colon of *S*. Typhimurium-infected mice (Fig. 4H). On the contrary, HNa pretreatment elevated colonic protein expression of M2 macrophage marker compared with the Mod group (*P *< 0.05). Next, in the Mod group, we observed that the mRNA and protein expression of toll-like receptor 4 (TLR4), myeloid differentiation primary response 88 (MyD88), inhibitor of kappa B kinase α (IKKα), nuclear factor kappa-B (NF-κB), and p-NF-κB exhibited a significant increase compared with those of the Con and HNa groups (*P *< 0.05), while HNa pretreatment profoundly reversed that (Fig. 4A and B) (*P *< 0.05). NOD-like receptor protein 3 (NLRP3) inflammasome has an important role in immune response and disease development as a component of intrinsic immunity. It has been well established that NLRP3 inflammasome can be activated via the NF-κB signaling pathway. The results revealed that *S*. Typhimurium infection significantly increased the protein expression of NLRP3, ASC, C-caspase1, GSDMD-N, Pro-IL-1β, and IL-18 in the colon of mice compared with that of the Con and HNa groups (*P *< 0.05). In line with expectations, HNa pretreatment abrogated these negative alterations (Fig. 4C and D).

Cluster of differentiation 4 (CD4^+^) and cluster of differentiation 8 (CD8^+^) T cells are primary immunocytes, which are involved in immune response and participate in intestinal mucosal immunity. In the present study, *S*. Typhimurium infection significantly decreased the protein expression of CD4^+^ T cells compared with the Con and HNa groups (*P < *0.05), while pretreatment with HNa significantly increased that (Fig. 4I). Importantly, CD4^+^ T cells could secrete IL-22, which can aid in intestinal resistance to bacterial infection. As shown in Fig. 4I, compared with that of the Mod group, the protein expression of IL-22, matrix metalloproteinase 7 (MMP7), toll-like receptor 9 (TLR9), and E-cadherin in the Tre group was remarkably upregulated (*P* < 0.05). Collectively, these results indicate that *S*. Typhimurium infection induced severe intestinal inflammation. It is worth noting that pretreatment with HNa alleviated intestinal inflammation by downregulating TLR4/NF-κB and NLRP3 signal pathways and modulating intestinal mucosal immunity.

### HNa improved intestinal barrier function.

**FIG 5:**
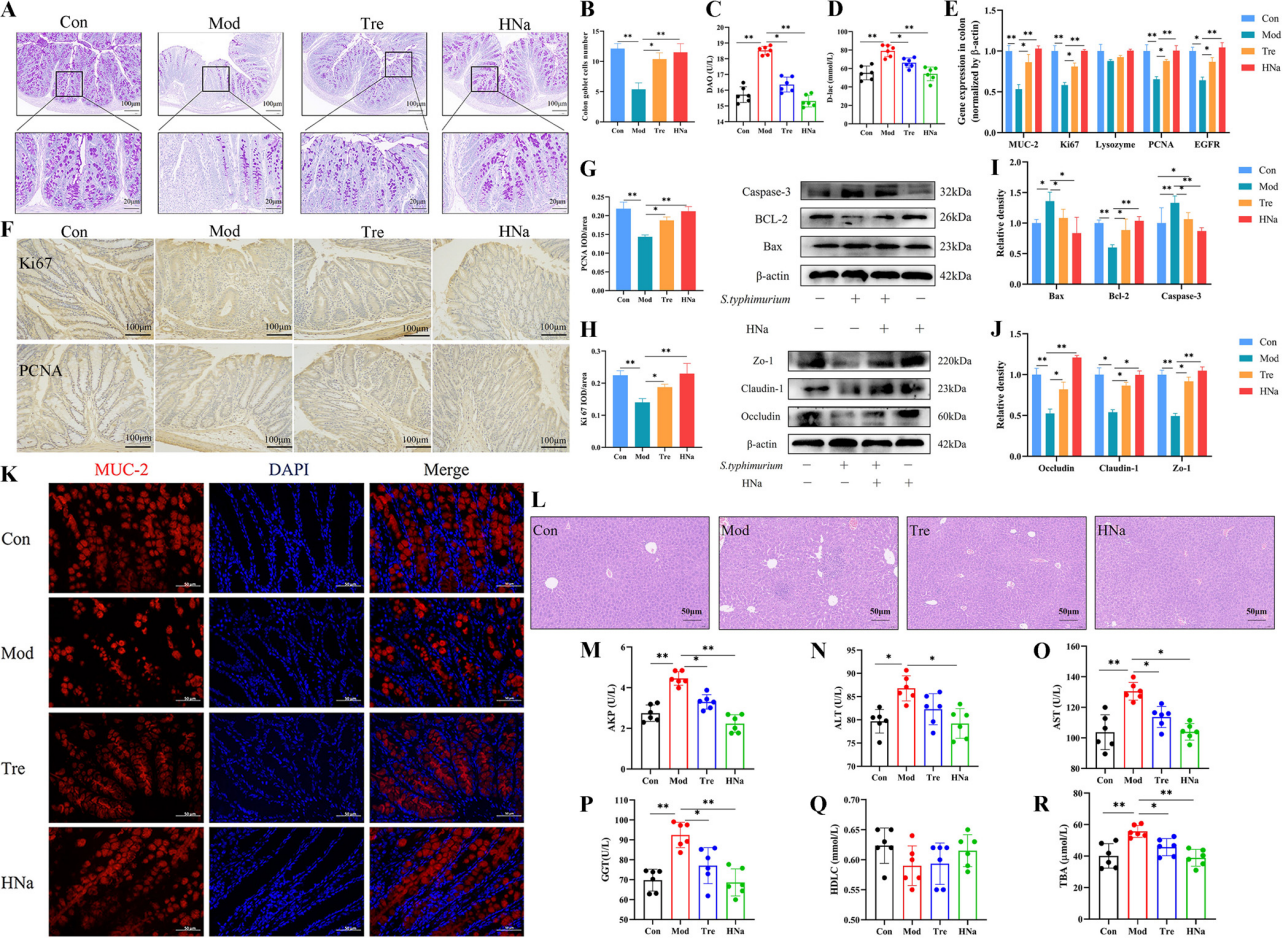
Administration of sodium humate (HNa) improved intestinal barrier function and alleviated liver damage in *Salmonella* Typhimurium-infected mice. Representative images of alcian blue-stained colonic sections (A) and the number of goblet cells (B). (C, D) The levels of serum diamine oxidase (DAO) and d-lactate (d-lac). (E) The mRNA expression of mucin-2 (MUC-2), Ki67, lysozyme, proliferating cell nuclear antigen (PCNA), and epidermal growth factor receptor (EGFR) in the colon. Microscopic images of colonic tissues stained with immunohistochemical of Ki67 and PCNA (F) and mean optical densities (G, H). (I) The protein expression of BCL2-associated X (Bax), B-cell lymphoma-2 (BCL-2), and caspase-3 in the colon. (J) The protein expression of occludin, claudin-1, and Zo-1 in the colon. (K) Relative expression of MUC-2 in colon tissues analyzed by immunofluorescence microscope. MUC-2 was stained as red, and nuclei are counterstained as blue. (L) H&E-stained liver sections. The levels of serum alkaline phosphatase (AKP) (M), alanine aminotransferase (ALT) (N), aspartate aminotransferase (AST) (O), gamma-glutamyl transferase (GGT) (P), high-density lipoprotein cholesterol (HDLC) (Q), and total bile acid (TBA) (R). Data were presented as means ± SD. Statistical significance was determined using one-way ANOVA, followed by Tukey test. *, *P* < 0.05; **, *P* < 0.01; ***, *P* < 0.001. Note that the results shown in Fig. 4A and in panel J are from the same experiment, and therefore, the blots labeled “β-actin” are identical.

Infection with pathogenic microorganisms leads to intestinal barrier dysfunction, which further increases intestinal permeability. As shown in Fig. 5J, *S*. Typhimurium infection significantly downregulated occludin, claudin-1, and Zo-1 protein expression in the colon compared with that in the Con group (*P *< 0.05). In contrast, HNa pretreatment significantly reversed *S*. Typhimurium-induced reduction of tight junction (TJ) protein expression. In addition, intestinal barrier dysfunction would result in an imbalance between proliferation and apoptosis in intestinal epithelial cells. We further verified the effects of HNa pretreatment on intestinal epithelial cell proliferation. The results showed that mice in the Tre group had higher mRNA expression of colon proliferating cell nuclear antigen (PCNA), epidermal growth factor receptor (EGFR), and Ki67 and higher protein expression of colon B-cell lymphoma-2 (BCL-2), PCNA, and Ki67 and lower protein expression of colon BCL2-associated X (Bax) and caspase-3 than those in the Mod group (*P *< 0.05) (Fig. 5E to I), indicating the protective effects of HNa on intestinal barrier function.

Intestinal barrier function is closely related to intestinal permeability; therefore, the number of colon goblet cells and the mRNA and protein expression of colon mucin-2 (MUC-2) were detected. The results showed that *S*. Typhimurium infection significantly decreased the numbers of goblet cells and the mRNA and protein expression of MUC-2 compared with those of the Con group (Fig. 5A and K) (*P *< 0.05). However, the decline in the Mod group was reversed by pretreatment with HNa. As shown in Fig. 5C and D, *S*. Typhimurium infection increased the levels of serum diamine oxidase (DAO) and d-lactate (d-lac), while HNa intervention ameliorated that (*P *< 0.05).

The impaired intestinal barrier function contributes to bacterial and bacteria-derived toxin translocation across the barrier, which can induce liver injury. In the present study, we found that the liver of *S*. Typhimurium-infected mice exhibited hepatocellular vacuolar change and heavy infiltration by mononuclear inflammatory cells, while these changes were ameliorated after the administration of HNa (Fig. 5L). As shown in Fig. 5M to R, serum levels of alkaline phosphatase (AKP), alanine aminotransferase (ALT), gamma-glutamyl transferase (GGT), and total bile acid (TBA) were markedly increased in *S*. Typhimurium-infected mice compared with those of the Con group but significantly decreased after HNa administration. Overall, pretreatment with HNa improved intestinal barrier function and reduced intestinal permeability, which in turn alleviated liver injury.

### Correlation analysis of microbiota and inflammatory and antioxidative mediator.

Correlation analysis of *S*. Typhimurium-infected mice with and without HNa administration revealed that 22 differentially bacterial genera had a significant correlation with the inflammatory factors and antioxidative mediators in the serum or colon (see Fig. S2). In detail, *g__Parabacteroides* was positively correlated with the levels of serum d-lac and colon IL-18 (*r* > 0.50; *P *< 0.05). *g__Salmonella* was positively correlated with the levels of serum DAO, MPO, IL-2, and MDA (*r* > 0.50; *P *< 0.05). *g__Flavobacterium* was positively correlated with colon level of IL-18 (*r* > 0.50; *P *< 0.05). *g__Cylindrospermopsis* was positively correlated with serum level of IFN-γ (*r* > 0.50; *P *< 0.05). *g__Bacteroides* was positively correlated with serum level of TNF-α (*r* > 0.50; *P < *0.05). *g__Mucispirillum* was positively correlated with the levels of serum IL-10, T-SOD, and colon sIgA (*r* > 0.50; *P *< 0.01). *g__Coprococcus* was positively correlated with the levels of serum IL-10, T-SOD, T-AOC, and GSH (*r* > 0.50; *P *< 0.01) and negatively correlated with the level of serum IL-2 (*r* > 0.50; *P *< 0.05). *g__Pseudanabaena* was positively correlated with the level of colon sIgA (*r* > 0.50; *P *< 0.05). *g__Lactobacillus* was positively correlated with the level of serum T-SOD (*r* > 0.50; *P* < 0.01).

### HNa-mediated gut microbiota alleviated intestinal damage and oxidative stress.

**FIG 6:**
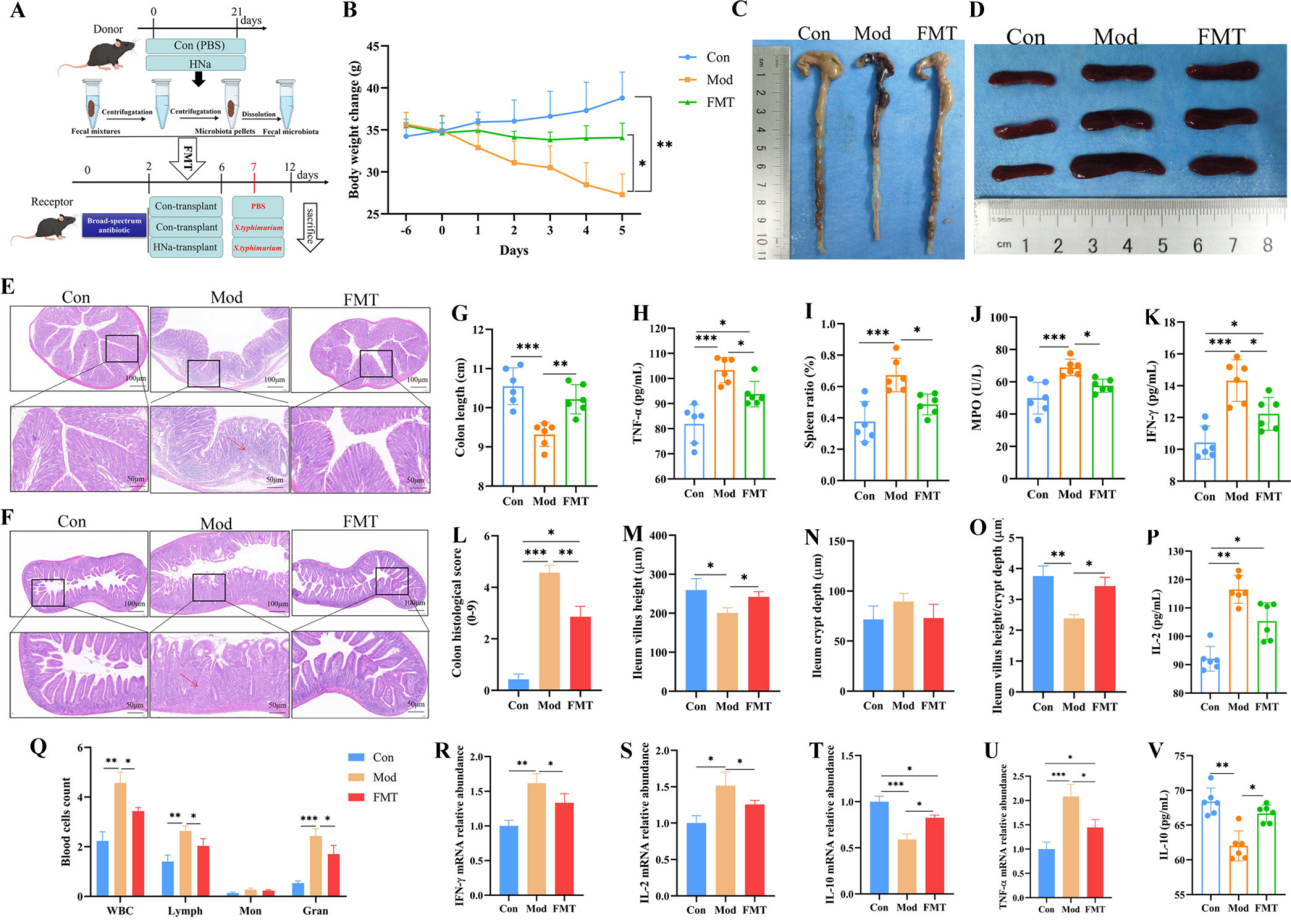
Sodium humate (HNa)-derived gut microbiota alleviated intestinal damage. (A) Diagram illustrating the animal experimental design employed in this section. (B) Daily body weight changes throughout the duration of the study. (C, G) The lengths of the colon. Macroscopic pictures of spleen (D) and spleen weight/body weight ratio (I). H&E-stained colon sections (E) and histological scores of colons (L). H&E-stained ileum sections (F) and villus height (M), crypt depth (N), and the ratio of villus height and crypt depth of ileum (O). (J) The activity of serum myeloperoxidase (MPO). The concentrations of tumor necrosis factor-α (TNF-α) (H), interleukin-2 (IL-2) (P), interferon-γ (IFN-γ) (K), and interleukin-10 (IL-10) (V) in the serum, and the mRNA expression (R to U) in the colon. (Q) Blood parameters of white blood cell (WBC), lymphocyte (Lymph), monocyte (Mon), and granulocyte (Gran). Data were presented as means ± SD. Statistical significance was determined using one-way ANOVA, followed by Tukey test. *, *P* < 0.05; **, *P* < 0.01; ***, *P* < 0.001.

To verify whether the HNa-mediated microbiota improves intestinal barrier function in *S*. Typhimurium-infected mice, we transplanted the fecal microbiota derived from mice receiving HNa gavage to *S*. Typhimurium-infected mice. As before, increased body weight (Fig. 6B), colon length (Fig. 6G), and ileal villus height (Fig. 6M) and decreased colon histological score (Fig. 6L) and spleen ratio (Fig. 6I) were shown in the FMT group compared with the Mod group (*P *< 0.05). As well, mice in the FMT group had lower levels of MPO, IL-2, IFN-γ, and TNF-α and higher levels of IL-10 in the serum as well as lower mRNA expression of IL-2, IFN-γ, and TNF-α and higher mRNA expression of IL-10 in the colon compared to that of the Mod group (*P* < 0.05) (Fig. 6R to U). In addition, FMT treatment significantly decreased the blood count of leucocytes, lymphocytes, and granulocytes (Fig. 6Q). Notably, FMT also increased the activities of T-AOC and CAT and decreased the concentration of MDA in serum compared to the Mod group (see Fig. S3A to E in the supplemental material).

### FMT regulated the composition of gut microbiota.

**FIG 7:**
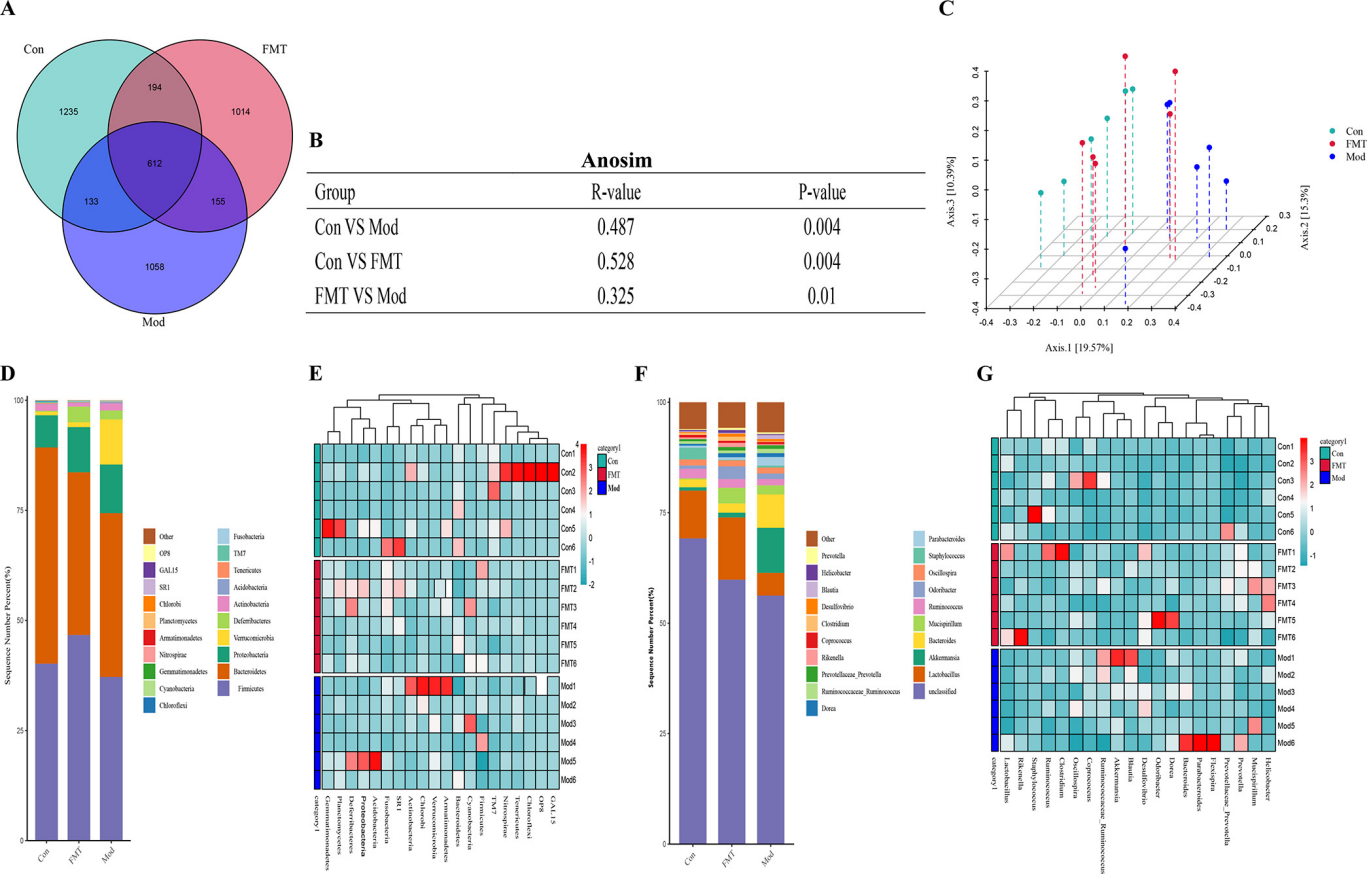
Sodium humate (HNa)-derived gut microbiota regulated the composition of gut microbiota. (A) A Venn diagram displayed the overlaps among groups. (B) ANOSIM among groups (*n* = 10). (C) Principal coordinates analysis (PCoA) plot of gut microbiota; each represented by one color (*n* = 10). Community bar plots and heatmap of phylum (D, E) and genus (F, G).

**FIG 8:**
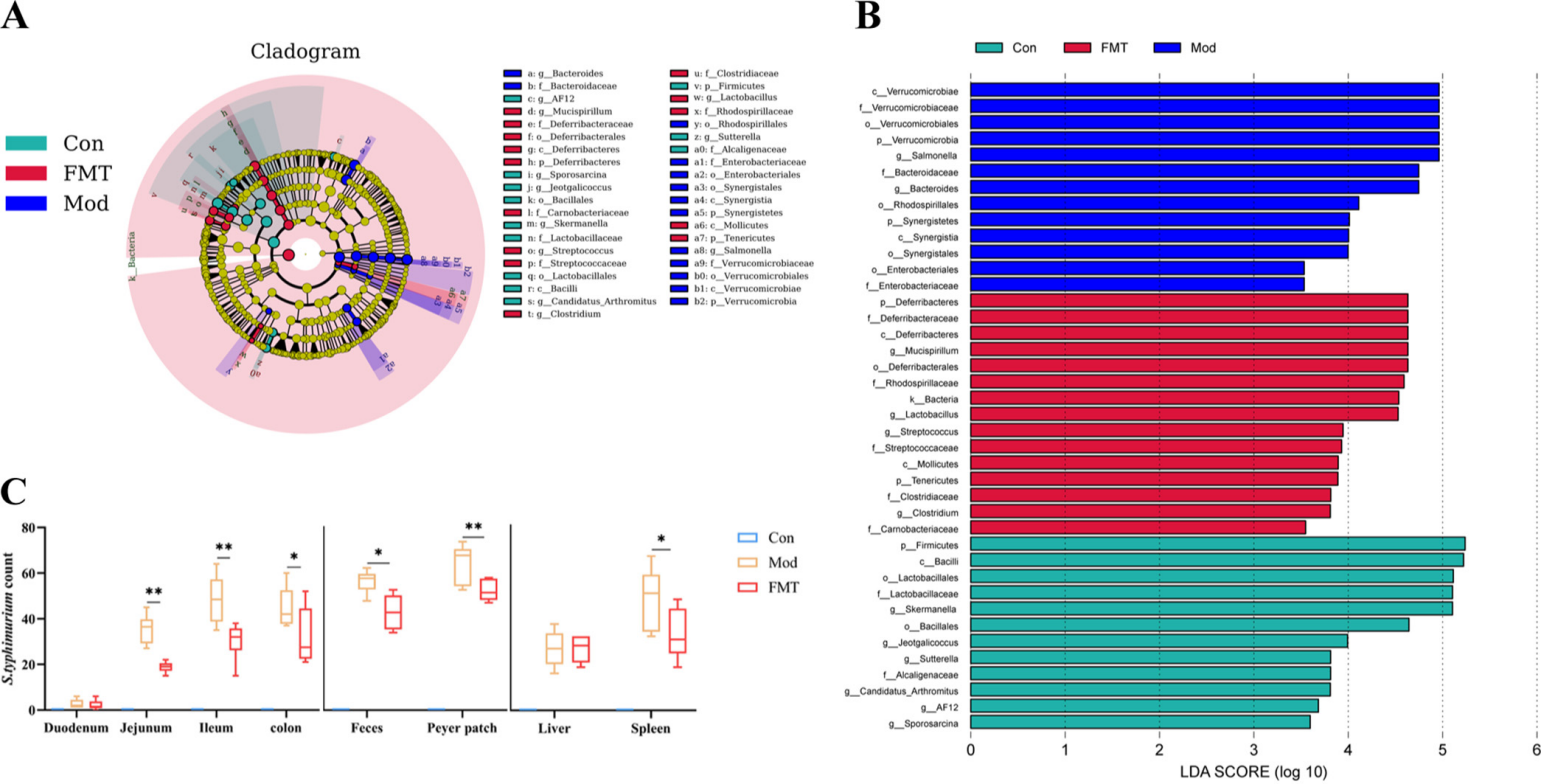
Sodium humate (HNa)-derived gut microbiota regulated the composition and function of gut microbiota. (A) Taxonomic cladogram of linear discriminant analysis effect size (LEfSe) analysis. Different colors indicate the enrichment of the biomarker taxa in different groups. The circle from inside to outside means the rank from kingdom to species, and the circle size represents the taxa abundance in the community. (B) Linear discriminant analysis (LDA) scores for different taxa abundances. (C) The number of *S*. Typhimurium in the duodenum, jejunal, ileal, colon and feces, payer patch, liver, and spleen (*n* = 10). Data were presented as means ± SD. Statistical significance was determined using one-way ANOVA, followed by Tukey test. *, *P* < 0.05; **, *P* < 0.01; ***, *P* < 0.001.

To further evaluate the impact of FMT on gut microbiota, the composition of gut microbiota was analyzed. Alpha diversity indexes were similar across groups (Fig. S3F to J). PCoA and ANOSIM analysis revealed the separation of microbiota structure between FMT and Mod groups (*R* = 0.325; *P = *0.01), which indicated that the gut microbiota structure was significantly influenced by FMT (Fig. 7B and C). The main composition in phylum levels was similar across groups (Fig. 7D). Of note, the genus *Mucispirillum*, *Lactobacillus*, *Clostridium*, and *Streptococcus* were significantly enriched by HNa-FMT and the genus *Bacteroides* and *Salmonella* were significantly enriched in the Mod group (Fig. 8A and B). Furthermore, we further examined the number of *S*. Typhimurium in mice feces, intestine, payer patch, liver, and spleen. As shown in Fig. 8C, the numbers of *S*. Typhimurium cells in the ileum, jejunum, colon, payer patch, spleen, and feces of *S*. Typhimurium-infected mice were significantly increased compared with those of the mice in the FMT group (*P *< 0.05). Taken together, the HNa-mediated gut microbiota also restored the balance of the gut microbiota in *S*. Typhimurium-infected mice.

### HNa-mediated gut microbiota alleviated intestinal inflammation.

**FIG 9:**
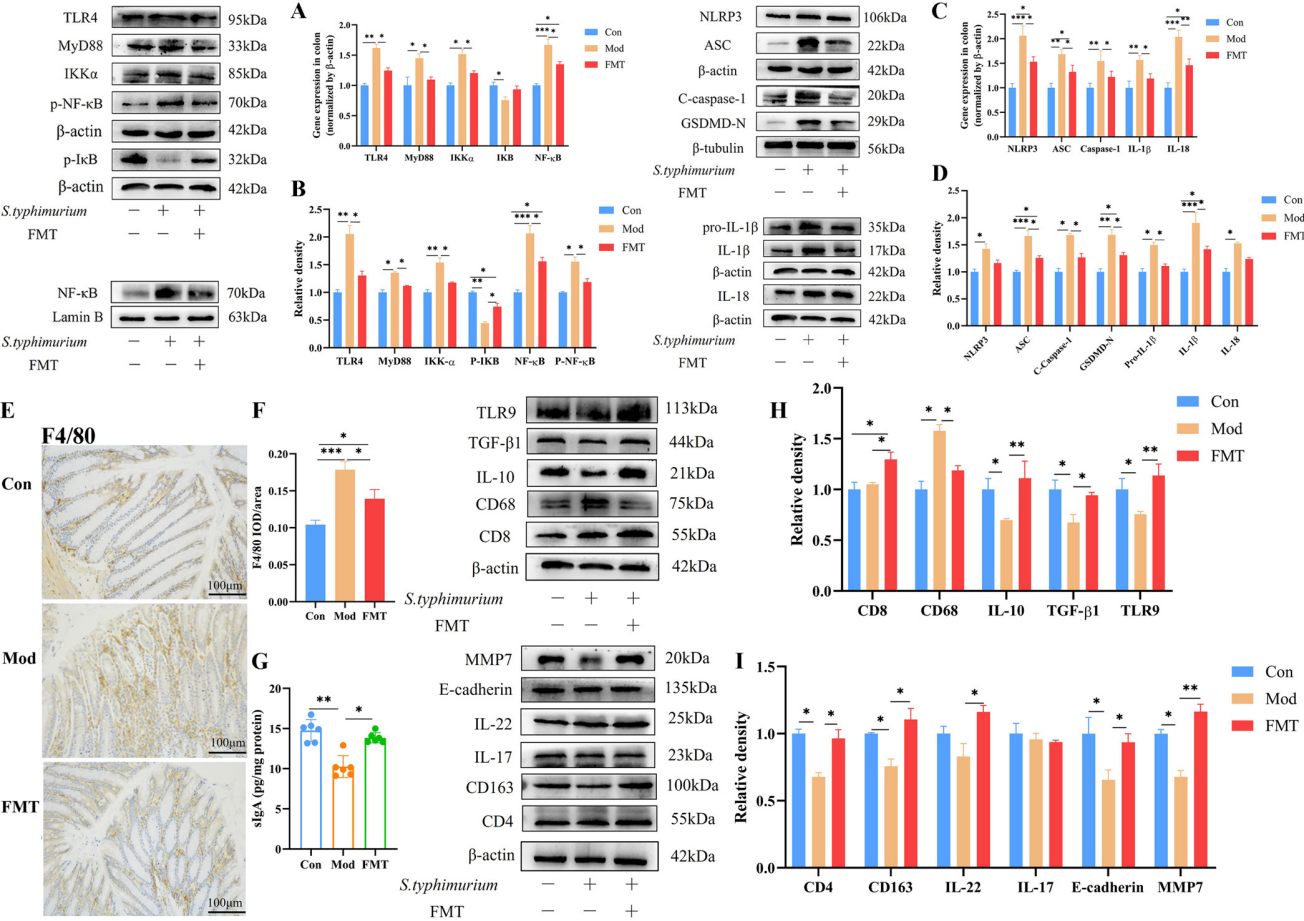
Sodium humate (HNa)-derived gut microbiota regulated the intestinal mucosa immunity and intestinal inflammation in *Salmonella* Typhimurium-infected mice. The mRNA (A) and protein (B) expression of TLR4/NF-κB signaling pathway-relevant proteins. The mRNA (C) and protein (D) expression of NOD-like receptor protein 3 (NLRP3) inflammasome-relevant proteins. Microscopic images of colonic tissues stained with immunohistochemical of F4/80 (E) and mean optical densities (F). (G) The concentration of secretory immunoglobulin A (sIgA) in the colon. (H) The protein expression of toll-like receptor 9 (TLR9), transforming growth factor-β1 (TGF-β1), interleukin-10 (IL-10), cluster of differentiation 68 (CD68), and cluster of differentiation 8 (CD8) in the colon. (I) The protein expression of matrix metalloproteinase 7 (MMP7), E-cadherin, interleukin-22 (IL-22), interleukin-17 (IL-17), cluster of differentiation 163 (CD163), and cluster of differentiation 4 (CD4) in the colon. Data were presented as means ± SD. Statistical significance was determined using one-way ANOVA, followed by Tukey test. *, *P* < 0.05; **, *P* < 0.01; ***, *P* < 0.001. Note that the results shown in panels A and C and panels A and D are from the same experiments, and therefore, the blots labeled “β-actin” are identical.

To investigate the regulatory effect of gut microbiota, we next validated the impact of HNa-derived microbiota on intestinal inflammation in *S*. Typhimurium-infected mice. The results showed that the HNa-derived microbiota improved the concentration of colon sIgA (Fig. 9G); upregulated the protein expression of M2-type macrophage markers CD163, IL-10, and TGF-β1; and downregulated M1 macrophage markers F4/80 and CD68 (*P *< 0.05). In addition, the expression of proteins related to the TLR4/NF-κB (TLR4, MyD88, IKKα, NF-κB, and p-NF-κB) and NLRP3 (ASC, C-caspase1, GSDMD-N, Pro-IL-1β, and IL-1β) signaling pathways revealed a significant decrease in the FMT group compared with the Mod group (*P *< 0.05). The results of mRNA expression were consistent with the expression of proteins (Fig. 9A to D). Notably, it could not be neglected that HNa-FMT profoundly increased the protein expression of CD4^+^ T and CD8^+^ T cells. Also, the protein levels of IL-22, MMP7, TLR9, and E-cadherin were increased by HNa-FMT compared with those of the Mod group (Fig. 9H and I). Collectively, these results suggested that HNa-mediated gut microbiota could regulate intestinal mucosal immunity and inhibit intestinal inflammation.

### HNa-mediated gut microbiota improved intestinal barrier function.

**FIG 10:**
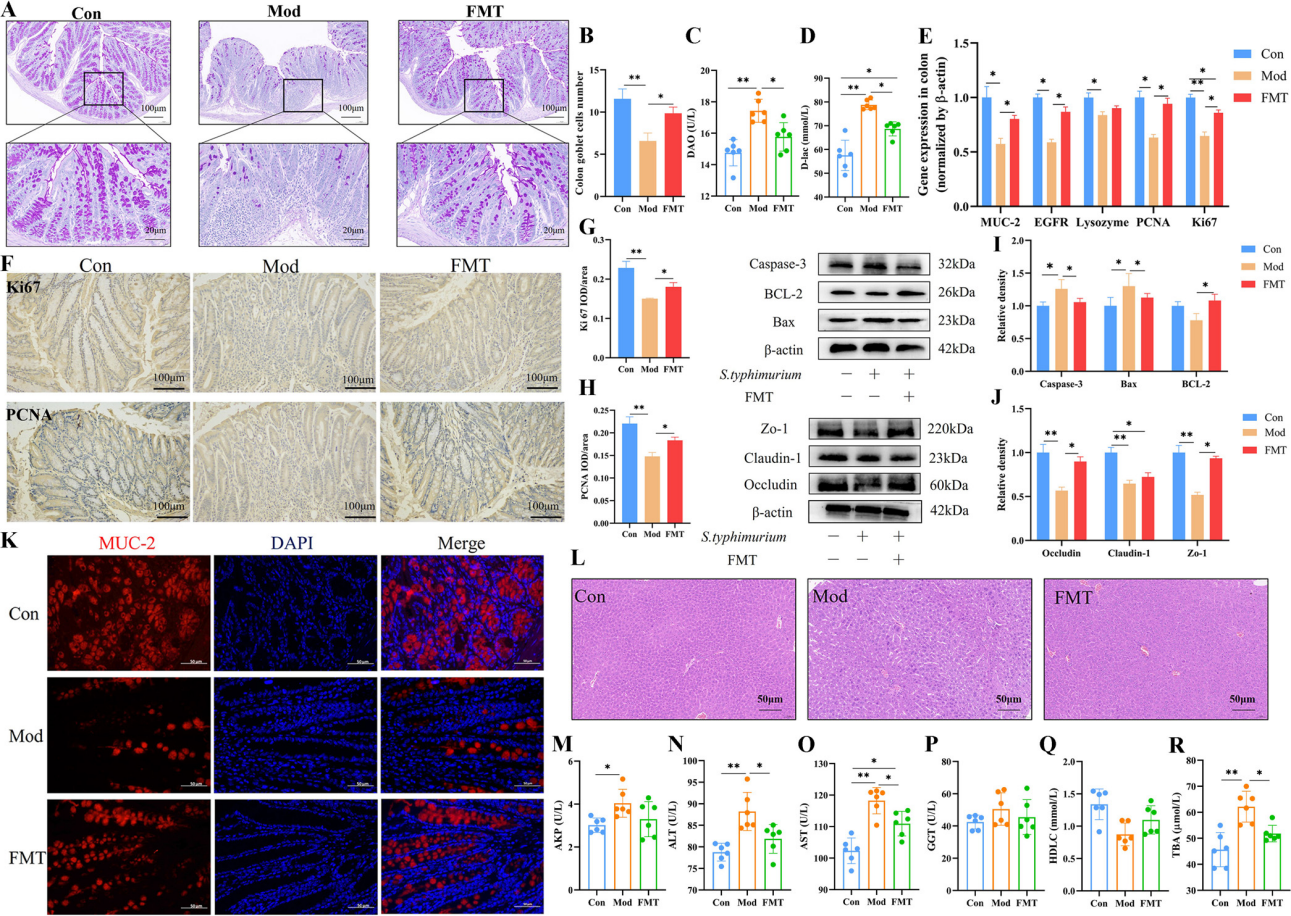
Sodium humate (HNa)-derived gut microbiota improved intestinal barrier function and alleviated liver damage in *Salmonella* Typhimurium-infected mice. Representative images of alcian blue stained colonic sections (A) and the number of goblet cells (B). (C, D) The levels of serum diamine oxidase (DAO) and d-lactate (d-lac). (E) The mRNA expression of mucin-2 (MUC-2), Ki67, lysozyme, proliferating cell nuclear antigen (PCNA), and epidermal growth factor receptor (EGFR) in the colon. Microscopic images of colonic tissues stained with immunohistochemical of Ki67 and PCNA (F) and mean optical densities (G, H). (I) The protein expression of BCL2-associated X (Bax), B-cell lymphoma-2 (BCL-2), and caspase-3 in the colon. (J) The protein expression of occludin, claudin-1, and Zo-1 in the colon. (K) Relative expression of MUC-2 in colon tissues analyzed by immunofluorescence microscope. MUC-2 was stained as red, and nuclei are counterstained as blue. (L) H&E-stained liver sections. The levels of serum alkaline phosphatase (AKP) (M), alanine aminotransferase (ALT) (N), aspartate aminotransferase (AST) (O), gamma-glutamyl transferase (GGT) (P), high-density lipoprotein cholesterol (HDLC) (Q), and total bile acid (TBA) (R). Data were presented as means ± SD. Statistical significance was determined using one-way ANOVA, followed by Tukey test. *, *P* < 0.05; **, *P* < 0.01; ***, *P* < 0.001.

To further analyze the effect of HNa-mediated gut microbiota on intestinal barrier function and intestinal permeability in *S*. Typhimurium-infected mice, we examined indicators related to intestinal tight junction protein, intestinal epithelial cell apoptosis and proliferation, and liver function. As shown in Fig. 10E to H, increased protein expression of occludin and Zo-1 and increased mRNA and protein expression of Ki67 and PCNA were observed in the FMT group compared with those of the Mod group (*P *< 0.05). Also, the FMT group had a higher BCL-2 protein expression and a lower Bax and caspase-3 protein expression compared with those of the Mod group (Fig. 10I). Meanwhile, HNa-derived gut microbiota increased the goblet cells number and the mRNA and protein expression of MUC-2 as compared with the Mod group (*P *< 0.05). Notably, the mice in the FMT group had lower levels of serum DAO and d-lac when compared to those of the Mod group (*P* < 0.05) (Fig. 10C and D). In summary, HNa-derived microbiota can maintain the intestinal barrier integrity of *S*. Typhimurium-infected mice by inhibiting apoptosis and promoting cell proliferation.

To verify whether the HNa-derived microbiota can alleviate liver injury induced by *S*. Typhimurium infection, we further analyzed the liver histological pathology and serum biochemical parameters related to liver inflammation. The results found that HNa-derived microbiota significantly alleviated the hepatocellular vacuolar change and heavy infiltration by inflammatory cells and decreased serum levels of AST, ALT, and TBA compared with those of the Mod group (*P *< 0.05) (Fig. 10M, N, and R). Overall, HNa-mediated gut microbiota also protected against mouse intestine damage from *S*. Typhimurium infection.

### Correlation analysis of microbiota and inflammatory and antioxidative mediator.

As shown in Fig. S3K, the correlation analysis found that *g__Bacteroides* showed a positive correlation with the levels of serum DAO, d-lac, IFN-γ, and MDA (*r* > 0.50; *P *< 0.05) but a negative correlation with colon sIgA level (*r* > 0.50; *P *< 0.05). *g__Salmonella* was positively correlated with the levels of DAO, IL-2, and MDA in the serum and IL-1β in the colon. *g__Mucispirillum* was positively correlated with the serum level of IL-10 and sIgA level in the colon (*r* > 0.50; *P *< 0.01) and negatively correlated with the levels of serum DAO, MPO, and IFN-γ (*r* > 0.50; *P *< 0.05). *g__Lactobacillus* was positively correlated with the serum levels of IL-10 and CAT (*r* > 0.50; *P *< 0.05).

## DISCUSSION

*Salmonella* Typhimurium can result in host enterocolitis, characterized by acute intestinal inflammation and diarrhea (22). Studies have revealed that *S*. Typhimurium is one of the main causes of foodborne illnesses worldwide (23). The antibacterial property of HNa has been widely reported (24). However, the antibacterial effect of HNa on *S*. Typhimurium is not clear. Here, we found that *S*. Typhimurium treated with sodium humate (HNa) appeared to have severe disruption of the cell wall and cell fragmentation. Importantly, the gene expression associated with *S*. Typhimurium motility, adhesion, and invasion, as well as cell wall integrity was significantly decreased by HNa treatment. Therefore, we confirmed the inhibitory effect of HNa on *S*. Typhimurium in *vitro*. To investigate the effect of HNa pretreatment on the intestinal *S*. Typhimurium and resident microflora, we used SS agar plates and MacConkey plates to isolate Escherichia coli and *S*. Typhimurium in the feces of HNa pretreatment mice. Interestingly, the result of scanning electron microscope (SEM) found no significant effect of HNa on the morphology of *S*. Typhimurium and E. coli. Importantly, gut microflora sequencing analysis also indicated that the diversity and abundance of intestinal microbiota were similar among groups. Thus, HNa does not disrupt the resident gut microflora. Taken together, we speculate that the antibacterial effects of HNa *in vivo* are mediated by increasing the abundance of beneficial bacteria or selectively inhibiting the proliferation of pathogenic bacteria. Thus, more studies are needed to investigate the antibacterial effects of HNa *in vivo*.

It is well known that gut microbiota is crucial for maintaining gastrointestinal tract homeostasis and host health. Dysbiosis of gut microbiota is one of the characteristics of intestinal diseases. Moreover, *S*. Typhimurium can exacerbate intestinal damage by disturbing the gut microbiota (20). In this study, we also discovered that the gut microbiota composition of *S*. Typhimurium-infected mice differed from that of controls. With the deepening of research on gut microbiota, therapeutic strategies for modulating gut microbiota, such as fecal microbiota transplantation (FMT), have attracted increasing attention. Importantly, our previous research found that administration with HNa can alleviate E. coli-induced intestinal damage by regulating gut microbiota and metabolites (11). Thus, we investigated whether HNa and HNa-derived microbiota alleviate intestinal damage caused by *S*. Typhimurium. In the present study, we also found that HNa regulated gut microbiota and enriched the abundance of beneficial bacteria such as *Mucispirillum* and *Coprococcus* in the intestinal tract of mice. Therefore, we speculate that the ameliorative effects of HNa on intestinal damage is closely related to the changed gut microbiota, and the results of FMT also proved that.

To explore how HNa attenuated the intestinal damage induced by *S*. Typhimurium, we performed the HNa pretreatment experiments. In line with previous research (6), the present study also found that *S*. Typhimurium could successfully colonize the intestinal tract of mice after 2 days of infection. The results found that HNa pretreatment alleviated *S*. Typhimurium-caused intestinal damage, as evidenced by the reduced colon shortening and colon and ileum histological damage. In agreement with the previous study (24), we confirmed that the administration of HNa decreased the proinflammatory cytokines such as myeloperoxidase (MPO), interferon-γ (IFN-γ), tumor necrosis factor-α (TNF-α), and interleukin-2 (IL-2) in both the serum and colon of *S*. Typhimurium-infected mice. Furthermore, some other typical indicators associated with *S*. Typhimurium infection, such as body weight loss and increased spleen index and blood leukocyte count, were also improved by HNa administration. In addition, the administration of HNa also attenuated *S*. Typhimurium infection-induced oxidative stress by increasing the activities of antioxidant enzymes and decreasing the content of malondialdehyde (MDA) in the serum.

Intestinal microecological balance contributes to intestinal homeostasis, mucosal immunity, and defense against pathogens (25). Numerous studies indicated that enteropathogenic microbiota infections such as E. coli, *Clostridioides*, and *S*. Typhimurium could perturb gut microbiota (26–28). The current study found that the gut microbiota structure was significantly different among groups. It was reported that dietary supplementation with HNa decreased the number of E. coli cells in piglets and mice challenged with enterotoxigenic Escherichia coli (ETEC) (11, 12). In this study, we found that pretreatment with HNa significantly impacted the structure of gut microbiota. As expected, HNa significantly enriched the abundance of beneficial bacteria, including *Mucispirillum*, *Coprococcus*, *Lactobacillus*, and *Odoribacter*. Moreover, the correlation analysis suggested that the altered bacteria by HNa, especially *Mucispirillum* and *Coprococcus*, were positively correlated with the anti-inflammatory cytokines and antioxidant enzymes. Herp et al. revealed that *Mucispirillum* protected mice against *S*. Typhimurium infection by interfering with pathogen invasion and virulence factor expression (29). Also, a recent study has shown that plant extract can modulate the liver expression of *miR-122* and *miR-34a* and alleviate nonalcoholic fatty liver by increasing the abundance of *Mucispirillum* (30). As is well known, *Coprococcus* is an intestinal core bacterium. It was reported that all microbes in genera *Coprococcus* can promote short-chain fatty acid (SCFA) production and have immunomodulatory and anti-inflammatory properties (31, 32). As the most common probiotic, *Lactobacillus* was reported to inhibit pathogen colonization, enhance mucosal immunity, and alleviate inflammation in the intestine (33). Consistently, our previous study also showed that administration with HNa increased the abundance of *Lactobacillus* (11). Collectively, these outcomes indicated that pretreatment with HNa contributed to a stable microecology. Moreover, the altered gut microbiota caused by HNa intervention could be crucial for maintaining intestinal homeostasis.

The body immune system is the first line of defense against invasion by pathogens (34). Innate and adaptive immune responses are linked by macrophages, which exert multifunctional characteristics, including secretion of cytokines, phagocytosis and killing of pathogens, and maintenance of immune homeostasis (35). In response to pathogenic infection, macrophages are differentiated into classically activated macrophages (M1) and alternatively activated macrophages (M2). Macrophages of the M1 type could mediate Th1 immune responses to phagocytosis and kill bacterial pathogens, which exert a crucial role in the initial stage of infection and acute phase of inflammation. M2 macrophages mediate Th2 immune responses responsible for tissue remodeling and homeostasis maintenance in the late phase of inflammation (36). In addition, as sentinels of innate immunity, toll-like receptors (TLRs) could participate in recognizing and defending against invading pathogens. To be specific, TLRs trigger the transcriptional activation of proinflammatory cytokines and chemokines, subsequently initiating defense mechanisms when recognizing pathogen ligands (37). Despite the fact that innate immune and inflammatory responses are crucial in the early stages of host defense against pathogen infection, the excessive inflammatory responses contribute to a cytokine storm and further cause intestinal damage (38). The roles of toll-like receptor 4 (TLR4) and its downstream transcription factor nuclear factor kappa-B (NF-κB) in mucosal immune responses have been extensively studied (39). It is well known that the activation of TLR4/MyD88/NF-κB signaling pathway may induce intestinal inflammation caused by pathogens (40). Specifically, it can promote the secretion of pro-IL-1β, which can be maturated via the activation of NOD-like receptor protein 3 (NLRP3) inflammasomes and further cleaved by caspase-1 (41). Numerous studies indicate that NF-κB activation could bind to the NLRP3 promoter in macrophages, and thus promote NLRP3 expression; moreover, pathogens can also directly induce the activation of NLRP3 inflammasome. Importantly, the overexpression of NF-κB and NLRP3 can increase the maturation and release of IL-1β and IL-18, which can further promote the activation of NLRP3 inflammasome and NF-κB via a feedback loop (38, 41, 42). Studies suggest a causal link between intestinal inflammation and macrophage differentiation in patients with inflammatory bowel disease (IBD), and further research found that inhibiting inflammatory signaling pathways and inducing polarization of macrophages to the M2 type have been considered as novel potential treatment approaches for IBD (43, 44). In addition, previous study indicated that polarizing the transformation of macrophages to the M2 type can protect against dextran sulfate sodium (DSS)-induced colitis (45). A latest study demonstrated that inhibition of NLRP3 inflammasome protected against septic intestinal damage (38). Herein, the roles of HNa on the intestinal alteration of M2 macrophages, the TLR4/NF-κB signaling pathway and NLRP3 inflammasome in *S*. Typhimurium-infected mice were explored. We observed that the expression of M1 macrophage marker F4/80, CD68, IL-1β, TLR4/NF-κB, and NLRP3 signaling pathway-associated proteins were notably upregulated in *S*. Typhimurium-infected mice, indicating that *S*. Typhimurium infection excessively activated the NF-κB and NLRP3 inflammasomes and promoted M1 macrophage polarization. As expected, pretreatment with HNa re-established the macrophage M1/M2 balance and suppressed the overexpression of TLR4/NF-κB signaling pathway and NLRP3 inflammasome in *S*. Typhimurium-challenged mice. Interestingly, we discovered that the protein expression of toll-like receptor 9 (TLR9) was upregulated in mice pretreated with HNa. It must be pointed out that the TLR9 deficiency in intestinal epithelial cells (IECs) led to hyperactivation of NF-κB and NLRP3 inflammasome signaling pathways, causing excessive secretion of inflammatory cytokines (46). In addition, the negative regulatory effect of TLR9 in IECs on the NF-κB/NLRP3/IL-1β pathway and the critical roles of TLR9 on the protection of intestinal integrity and defense against *S*. Typhimurium infection were well demonstrated (37).

As the primary effector cells of intestinal mucosal humoral immunity, secretory immunoglobulin A (sIgA) is of great importance in the host intestinal defense against pathogen infection. It was reported that sIgA can influence the composition of gut microbiota by inhibiting or promoting the growth of specific taxa and also prevent pathogenic bacteria from invading the intestine. In addition, sIgA-deficient mice presented severe dysbiosis and chronic gut inflammation compared with wild-type mice (47). Here, we found that pretreatment with HNa markedly increased the concentration of sIgA in the colon of mice in comparison with that of *S*. Typhimurium-infected mice. Thus, we speculate that the changed gut microbiota of mice in the Tre group may be related to the increased sIgA. T lymphocytes are important immune cells in the body immune system, which are mainly divided into CD4 (helper T cells [Th]) and CD8 (cytotoxic T cells [Tc]) T cells. CD4 T cells can further turn into different effector cells to perform multiple immunological functions when antigen stimulation occurs (48). Consistent with previous studies (49), *S*. Typhimurium infection decreased the levels of intestinal CD4 and CD8 T cells. In keeping with our previous study (11), the present study also indicated that pretreatment with HNa upregulated the levels of the colon CD4 and CD8 T cells in *S*. Typhimurium-infected mice. Furthermore, some researchers have demonstrated that intestinal microflora such as *Lactobacillus* and their products can stimulate group 3 innate lymphoid cells (ILC3) and CD4 T cells to produce IL-22 (50). It is generally known that IL-22, particularly IL-22 secreted by CD4 T cells, plays a more important role on the defense against bacterial infection and the protection of intestinal crypt epithelial cells (51). It was reported that IL-22 not only promoted goblet cells to secrete mucus but also promoted intestinal epithelial cell proliferation by increasing the protein expression of intestinal epithelial cell matrix metalloproteinases 7 (MMP7) and E-cadherin (52). This study found that the levels of colon CD4 T cells, IL-22, MMP7, and E-cadherin were upregulated by HNa pretreatment in *S*. Typhimurium-infected mice compared with mice in the Mod group. Above all, pretreatment with HNa also upregulated the protein expression of anti-inflammatory cytokines IL-10 and transforming growth factor-β (TGF-β). The above-mentioned results further confirmed the immunomodulatory effects of HNa.

The intestine is an important organ that maintains the immunity and nutrition of the body. An integrated intestinal mucosal barrier maintains the host immunity as well as prevents pathogenic bacteria and toxins from damaging the intestines (53). Intestinal barrier function is primarily reflected by the integrity of intestinal epithelial cell barrier, which consists of IECs and intercellular tight junctions (TJs). The TJ proteins such as Zo-1, claudin-1, and occludin are responsible for the diffusion of intestinal epithelium and among cells (53). The secretion of mucin 2 (MUC2) into the intestinal lumen by goblet cells creates the first line of defense against pathogen encroachment. In addition, antimicrobial peptides (AMPs) such as lysozyme secreted by IECs further reinforced intestinal barrier function (54). It was reported that *S*. Typhimurium infection can damage intestinal barrier by thinning the mucus layer and decreasing the expression of TJ proteins (22). In previous studies, the reduced mucus layer thickness was also associated with pathogen infection (55). Similarly, the results of the current study showed that *S*. Typhimurium infection decreased the counts of goblet cells, the mRNA and protein abundance of MUC2, and the protein expression of occludin, claudin-1, and Zo-1 in the colon of mice, while pretreatment with HNa effectively attenuated *S*. Typhimurium infection-induced intestinal barrier damage. It has been reported that *S*. Typhimurium-induced intestinal damage is closely related to its promotion of IEC apoptosis (19). In agreement with that, we found that the pro-apoptotic proteins BCL2-associated X (Bax) and caspase-3 were significantly upregulated while the anti-apoptotic protein B-cell lymphoma-2 (BCL-2) was significantly downregulated in *S*. Typhimurium-infected mice. Moreover, HNa was shown to repair wound healing by promoting proliferation, migration, and angiogenesis of epithelial cells (10). Intestinal barrier integrity can be measured by serum activity of DAO and concentration of d-lac. The results of our study indicated that HNa pretreatment can maintain intestinal barrier integrity in *S*. Typhimurium-infected mice, which was reflected by the decreased levels of DAO and d-lac in the serum. *S*. Typhimurium infection can damage intestinal barrier, while increased intestinal permeability contributes to bacterial and bacteria-derived toxin translocation across the barrier, which can induce liver injury (56). Here, we found that the liver of *S*. Typhimurium-infected mice exhibited hepatocellular vacuolar change and mononuclear cell infiltration. Also, the serum biochemical indicators alkaline phosphatase (AKP), alanine aminotransferase (ALT), gamma-glutamyl transferase (GGT), and total bile acid (TBA) reflecting liver injury were markedly increased in *S*. Typhimurium-infected mice. All of the above results demonstrated that *S*. Typhimurium infection could result in liver damage. Thus, we speculate that severe liver damage caused by *S*. Typhimurium infection may be associated with gut microbiota disturbance and increased intestinal permeability. Importantly, pretreatment with HNa alleviated hepatocellular vacuolar and inflammatory cell infiltration caused by *S*. Typhimurium infection.

We have previously demonstrated that HNa had favorable effects on intestinal barrier function through modifications of gut microbiota and metabolite composition (11). Similarly, we discovered that HNa pretreatment altered gut microbiota composition, namely, enriching bacteria such as *Coprococcus*, *Lactobacillus*, and *Mucispirillum*, and may contribute to promote an anti-inflammatory state in the intestine. However, no study used HNa-derived gut microbiota as a donor for FMT to repair intestinal damage caused by *S*. Typhimurium infection. Therefore, we next further validated these probiotic effects of HNa-derived gut microbiota by FMT. According to the results, FMT significantly influenced microbial community structure. As expected, FMT also significantly increased the abundance of *Mucispirillum*, *Clostridium*, and *Lactobacillus*. Importantly, HNa-derived gut microbiota dramatically abolished the *S*. Typhimurium-induced intestinal damage, including changes in body weight change, colon length, spleen index, histological injury of the ileum and colon, and inflammatory cytokines expression. Interestingly, we found that intestinal damage was aggravated in mice pretreated with broad-spectrum antibiotics. We speculate that the reason for that is attributed to antibiotic pretreatment disrupting the intestinal flora and further promoting the colonization of *S*. Typhimurium. In addition, HNa-derived gut microbiota suppressed the TLR4/MyD88/NF-κB signaling pathway and NLRP3 inflammasome in the colon of *S*. Typhimurium-infected mice by promoting macrophage polarization into M2 type and increasing the levels of intestinal CD4 T and CD8 T cells and sIgA. Importantly, FMT upregulated the colonic secretion of anti-inflammatory cytokines such as IL-10, TGF-β, and TLR9 compared with those of the mice in the Mod group, which further inhibited intestinal inflammatory responses induced by *S*. Typhimurium infection. We next strive to ascertain the role of HNa-derived gut microbiota on intestinal barrier function in *S*. Typhimurium-infected mice and the impact of gut microbiota on the proliferation and apoptosis of IECs. Consistent with that of HNa itself, HNa-derived gut microbiota downregulated the expression of pro-apoptotic proteins Bax and caspase-3 and upregulated the expression of promoted IECs proliferation proteins such as IL-22, MMP7, E-cadherin, Ki67, and PCNA, suggesting that intestinal damage is primarily alleviated by HNa-derived gut microbiota. Also, the increased number of goblet cells and protein abundance of MUC-2, occludin, claudin-1, and Zo-1 in the colon and reduced serum levels of DAO and d-lac were detected in mice with FMT, which further confirmed that gut microbiota is closely associated with intestinal barrier function. It is well known that the liver is in close contact with the intestine, and changes in the gut microbiota affect that (57). It was reported that the intestinal microbiome influences liver immune functions through a “gut-microbiome-liver” axis (25). Here, we found that HNa-derived gut microbiota alleviated liver injury and decreased the levels of serum ALT, AST, and TBA, and we speculate that the effects of protecting the liver may be attributed to its protective effects on intestinal barrier function. Collectively, our results suggested that HNa-derived gut microbiota, particularly *Lactobacillus* and *Mucispirillum* enrichment, played a key role in alleviating intestinal damage caused by *S*. Typhimurium infection. These increased microbes induced by HNa also contribute to maintaining intestinal barrier function by improving IEC proliferation, regulating mucosal immunity, and inhibiting the intestinal inflammatory response.

In summary, this study revealed that pretreatment with HNa alleviated *S*. Typhimurium infection-induced intestinal barrier damage by increasing the abundance of gut beneficial bacteria, modulating intestinal mucosal immunity, inhibiting intestinal inflammatory response, and promoting IEC proliferation. Moreover, we firstly confirmed that FMT of HNa-derived microbiota could alleviate *S*. Typhimurium infection-induced intestinal damage and protect the intestinal barrier function. These findings demonstrated the protective role of HNa and HNa-derived gut microbiota in intestinal health, which contributes to the development of therapeutic and preventive strategies for *S*. Typhimurium and other intestinal pathogen infections in humans and animals.

## MATERIALS AND METHODS

Sodium humate (HNa) was purchased from the Institute of Coal Chemistry, Chinese Academy of Sciences (Shanxi, China). *Salmonella* Typhimurium was obtained by Harbin Veterinary Research Institute, CAAS. *S*. Typhimurium was cultured in Luria-Bertani broth and incubated at 37°C for 12 h, and then centrifuged at 8,000 rpm for 10 min. Bacteria were washed three times with phosphate-buffered saline (PBS). Finally, concentration of *S*. Typhimurium was adjusted to 1 × 10^7^ CFU/mL by PBS.

### Animals and experimental design.

The experimental protocol was approved by the Ethics Committee of Northeast Agricultural University (Harbin, China). Specific pathogen-free (SPF) female mice were purchased from Chang Sheng Biotechnology Co., Ltd. (Liaoning, China). The mice were kept on a 12 h light/dark cycle with constant temperature and humidity under pathogen-free conditions. All mice had free access to food and water throughout the experimental period. After 7 days of adaption, a total of 30 female mice were randomly distributed to five groups (*n* = 6) to ascertain the optimal protective concentration of HNa against *S*. Typhimurium in mice, including the Con, Mod, 0.1% HNa, 0.3% HNa, and 0.5% HNa groups. The mice in the Con and Mod group were gavaged 0.2 mL of PBS daily, while the mice in the HNa groups were, respectively, gavaged 0.2 mL of 0.1%, 0.3%, and 0.5% HNa daily. The mice in the Mod, 0.1%, 0.3%, and 0.5% HNa groups were given 0.2 mL of 1 × 10^7^ CFU/mL *S*. Typhimurium, intragastrically administered on day 1. Monitoring and recording of mouse survival were done daily for 10 days.

In another trial, a total of 24 female mice were randomly assigned to four groups (*n* = 6), including the Con, Mod, Tre, and HNa groups (Fig. 1A). The trial lasted for 26 days. Prior to *S*. Typhimurium infection, mice were fasted for 12 h and banned water for 4 h. The mice in the Con and Mod group were gavaged 0.2 mL of PBS daily, while Tre and HNa group mice were gavaged 0.2 mL of 0.5% HNa daily (the optimum concentration of HNa was obtained from the previous part of this study). The mice in the Mod and Tre groups were given 0.2 mL of 1 × 10^7^ CFU/mL *S*. Typhimurium, intragastrically administered on day 22 (6). The body weight of each mouse was recorded before and after *S*. Typhimurium infection. On day 27, blood samples were collected from the orbital venous plexus and distributed to two vacuum tubes, one containing anticoagulant agent and the other without anticoagulant agent for subsequent analysis. Afterwards, the mice were sacrificed by cervical dislocation, and the colon length was measured and the spleen weight was obtained for calculating the spleen index as follows: spleen index (%) = (spleen weight/body weight) × 100%. The liver and the middle segments (approximately 1 cm) of the ileum and colon were fixed in 4% paraformaldehyde solution for histomorphology analysis. The remaining colon samples were immediately frozen in liquid nitrogen and stored at −80°C for subsequent analysis. Fresh feces were collected and stored at −80°C for sequencing analysis of microbiota.

Fecal microbiota transplantation (FMT) experiment. A total of 12 female donor mice were randomly divided into two groups, Con donors and HNa donors. The Con donor group was gavaged 0.2 mL of PBS, while the HNa donor group was gavaged 0.2 mL of 0.5% HNa daily for 21 days. Fresh feces from each mouse were collected, weighed, homogenized, and suspended in PBS. The samples were then centrifuged, and the supernatant was filtered and used for FMT treatments (58).

A total of 18 female receptor mice were randomly distributed into three groups (*n* = 6) for the FMT experiment (Fig. 6A). After 1 week of broad-spectrum antibiotic treatment (50 μg/mL penicillin, 50 μg/mL clindamycin, 50 μg/mL neomycin, 50 μg/mL metronidazole, and 25 μg/mL vancomycin) (Sigma, St. Louis, USA) in drinking water (59), the mice in the Con and Mod groups were administered fecal microbiota from the Con donors and those in the FMT group were given fecal microbiota from the HNa donors. On day 7, the mice of Mod and FMT groups were orally gavaged with 0.2 mL of 1 × 10^7^ CFU/mL *S*. Typhimurium, and the mice in the Con group were orally gavaged with 0.2 mL PBS (6). On day 12, the mice were sacrificed, and the sample collection procedure is the same as mentioned above.

### Effects of HNa on *S*. Typhimurium morphology and the gene expression of virulence, motility, and colonization *in vitro*.

*S. Typhimurium* was cultured in LB broth for 12 h at 37°C before being adjusted to 1 × 10^7^ CFU/mL with PBS. Approximately 50 μL of the dilution was incubated on LB agar plates containing 0.5% HNa for 24 h at 37°C. For morphology characterization, the bacteria were harvested and then dehydration with ethanol (60). Morphological properties of *S*. Typhimurium were determined by scanning electron microscope (SEM) (Hitachi S-4800).

The monoclonal colony was picked and inoculated in LB medium, and then cultured in shaker at 37°C until optical density at 600 nm (OD_600_) = 1.0 (~10^9^ CFU/mL). The bacterial cells were washed three times with PBS, and total RNA was extracted using the RNeasy minikit (Qiagen, Germantown, MD, USA), and then reverse transcribed into cDNA using the iScript reverse transcriptase kit. The StepOnePlus platform was used for qRT-PCR. The relative gene expression was calculated with the 2^−ΔΔ^*^CT^*. The primers (see Table S1 in the supplemental material) are referenced from previously published literature (61) and commercially synthesized by Kumei Biotechnology (Jilin, China).

### *S.* Typhimurium colonization.

The fresh feces were collected for bacterial counting to reflect the colonization of *S*. Typhimurium at 2 days postchallenge.

The mice were sacrificed 4 days postchallenge, and the fresh feces, intestine tissue, payer patch, spleen, and liver were collected and suspended in PBS. Ten-fold serial dilutions were then plated on SS agar plates (Oxoid, Hampshire, UK). After that, the SS agar plates were incubated at 37°C for 24 h. The results are presented as log_10_ CFU/mg. All counts were performed in triplicate.

### Histomorphological analysis.

Tissue samples of the liver, ileum, and colon fixed with 4% paraformaldehyde were embedded in paraffin and then cut into 5 μm-thick sections, which were stained with hematoxylin-eosin (H&E) or alcian blue. The tissue damage was examined using light microscopy (Olympus, Tokyo, Japan), and the goblet cells, villus height, and crypt depth were measured with Image-Pro Plus 6.0 software (Media Cybernetics, MD, USA). A validated scoring system was used to evaluate colon pathology by a single-blind scorer (62).

### Enzyme-linked immunosorbent assay.

The levels of serum myeloperoxidase (MPO), interleukin-2 (IL-2), interleukin-10 (IL-10), interferon-γ (IFN-γ), tumor necrosis factor α (TNF-α), and colon secretory immunoglobulin A (sIgA) were measured using enzyme-linked immunosorbent assay (ELISA) kits (Jingmei Biotechnology Co., Ltd, Jiangsu, China). The levels of serum high-density lipoprotein cholesterol (HDLC), total bile acid (TBA), d-lactate (d-lac), diamine oxidase (DAO), alkaline phosphatase (AKP), alanine aminotransferase (ALT), gamma-glutamyl transferase (GGT), aspartate aminotransferase (AST), glutathione (GSH), catalase (CAT), total antioxidant capacity (T-AOC), total superoxide dismutase (T-SOD), and malondialdehyde (MDA) in the serum were analyzed according to the manufacturer’s instructions (Nanjing Jiancheng Co., Ltd, Nanjing, China).

### Immunohistochemistry.

Paraffin-embedded slides from colon tissue were firstly deparaffinized using dimethyl benzene and graded ethanol, then blocked with 5% bovine serum albumin (BSA), and stained with primary antibodies overnight at 4°C as follows: Ki67, F4/80, and proliferating cell nuclear antigen (PCNA) (1:200; Wanlei Biotechnology, Liaoning, China). This was followed by staining with horseradish peroxidase (HRP)-labeled goat anti-rabbit IgG secondary antibodies for 20 min. Sections were dyed with diaminobenzidine (DAB) substrate color liquid after washing. Images were analyzed using Image-Pro Plus 6.0 software (Media Cybernetics, MD, USA).

### Immunofluorescence.

The deparaffinized colon tissue sections were soaked in sodium citrate buffer for antigen repair. After blocking with 3% BSA, the sections were overnight incubated with the primary antibody (Mucin-2; ABcolonal, Wuhan, Hubei, China). After washing, the sections were incubated with fluorescent secondary antibodies. DAPI (4′,6-diamidino-2-phenylindole) was used to identify the cell nucleus, and the sections were examined by fluorescence microscopy (Nikon Eclipse C1; Nikon, Japan).

### Real-time PCR for gene expression analysis.

The Eastep Super Total RNA extraction kit (Promega Biotech Co, Ltd, Beijing, China) was used to collect the total RNA from the colon, then reverse transcribed into cDNA using the PrimeScript RT reagent kit and genomic DNA (gDNA) Eraser kit (TaKaRa Biomedical Technology, Beijing, China). The Roche 480 System (Roche, Switzerland) was used for qRT-PCR. The relative mRNA abundance was calculated with the 2^−ΔΔ^*^CT^*. The qRT-PCR primers (see Table S2 in the supplemental material) were commercially synthesized by Kumei Biotechnology (Jilin, China).

### Western blotting.

Protease inhibitor-containing radioimmunoprecipitation assay (RIPA) lysis buffer was used to homogenize colon tissues. The protein concentration was measured with a bicinchoninic acid (BCA) protein assay kit (Beyotime Biotechnology, Shanghai, China). Equal amounts of protein were electrophoresed on SDS-PAGE gels and then transferred to 0.22-μm or 0.45-μm polyvinylidene difluoride (PVDF) membranes. The membranes were blocked with 5% skimmed milk for 2 h and then incubated with primary antibodies overnight. The membranes were washed with Tris-buffered saline containing Tween 20 (TBST) and incubated with secondary antibodies for 1 h at room temperature. Enhanced chemiluminescence (ECL) reagents (Tanon, Shanghai, China) were used to detect the expression of target proteins and quantified using Image J software.

### Gut microbiota analysis.

Total DNA was extracted from fecal samples of mice. Hypervariable V3-V4 regions of bacterial 16S rRNA gene were amplified by PCR specific with 338F-806R primers, and the PCR products were sequenced by the NovaSeq platform (Illumina, San Diego, USA). Microbiome bioinformatics was performed with QIIME 2 (version 2019.4). With the demux plugin, raw sequence data were demultiplexed followed by primers being cut with the cutadapt plugin. DADA2 was used to quality filter, denoise, merge, and remove chimeras from the sequences. In order to construct a phylogeny with fasttree2, nonsingleton amplicon sequence variants (ASVs) were aligned with Mafft. Alpha diversity and beta diversity were calculated by random normalization to the same sequences. Taxonomy was assigned to ASVs using the classify-sklearn naïve Bayes taxonomy classifier. Sequences with 100% similarity were assigned to the same operational taxonomic unit (OTUs). The statistical difference in the relative abundance of microbiota among groups was compared using the linear discriminant analysis (LDA) and linear discriminant analysis effect size (LEfSe).

### Statistical analysis.

All of the data were presented as mean ± standard deviation (SD). The statistical differences were analyzed by one-way analysis of variance (ANOVA) (SPSS 22, Inc., IBM, Chicago, USA) followed by Turkey's multiple comparison test. LDA scores of >3.5 and a *P* value of <0.05 were denoted as significant differences between microbial genera. Statistical significance was indicated as *P *< 0.05. The correlation analysis was estimated by Spearman’s correlation coefficient. Correlations were considered significantly different at *r* > 0.50 or *r* < −0.50 and *P *< 0.05.

### Data availability.

The data of 16S sequencing are openly available in NCBI under BioProject accession numbers PRJNA913279 and PRJNA913402.
