# Supplementary material for: Sodium Humate-Derived Gut Microbiota Ameliorates Intestinal Dysfunction Induced by Salmonella Typhimurium in Mice
Source: Microbiol Spectr. 2023 Apr 17;11(3):e05348-22. doi: 10.1128/spectrum.05348-22 (PMC10269575; doi:10.1128/spectrum.05348-22)
Supplement: Supplemental file 1 — Supplemental material. Download spectrum.05348-22-s0001.pdf, PDF file, 0.4 MB [file spectrum.05348-22-s0001.pdf]

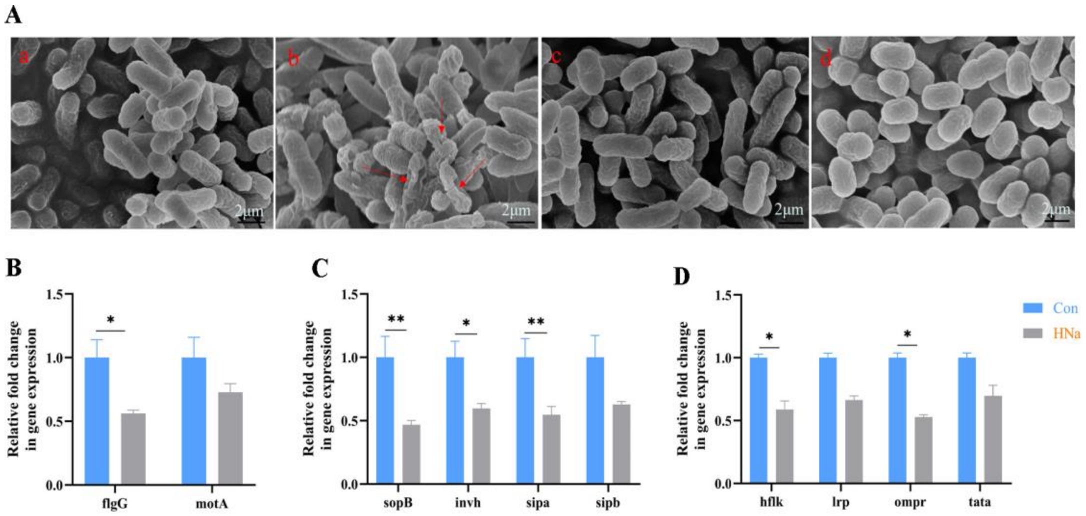

FIG S1 Sodium humate (HNa) disrupted the cell structure of S. Typhimurium and decreased the gene expression of virulence. (A) Representative SEM images of bacteria (a: S. Typhimurium without HNa treated in vitro; b: S. Typhimurium treated with HNa in vitro; c: S. Typhimurium isolated from the feces of mice pretreatment with HNa; d: *E. coli* isolated from the feces of mice pretreatment with HNa). The genes expression associated with S. Typhimurium (B) motility (flgG, motA), (C) adhesion and invasion (sopB, invh, sipa, and sipb), and (D) cell membrane and cell wall integrity (hflk, lrp, ompr, and tata). Statistical significance was determined using one-way ANOVA, followed by Tukey test. $^{*}P < 0.05$, $^{**}P < 0.01$, $^{***}P < 0.001$.

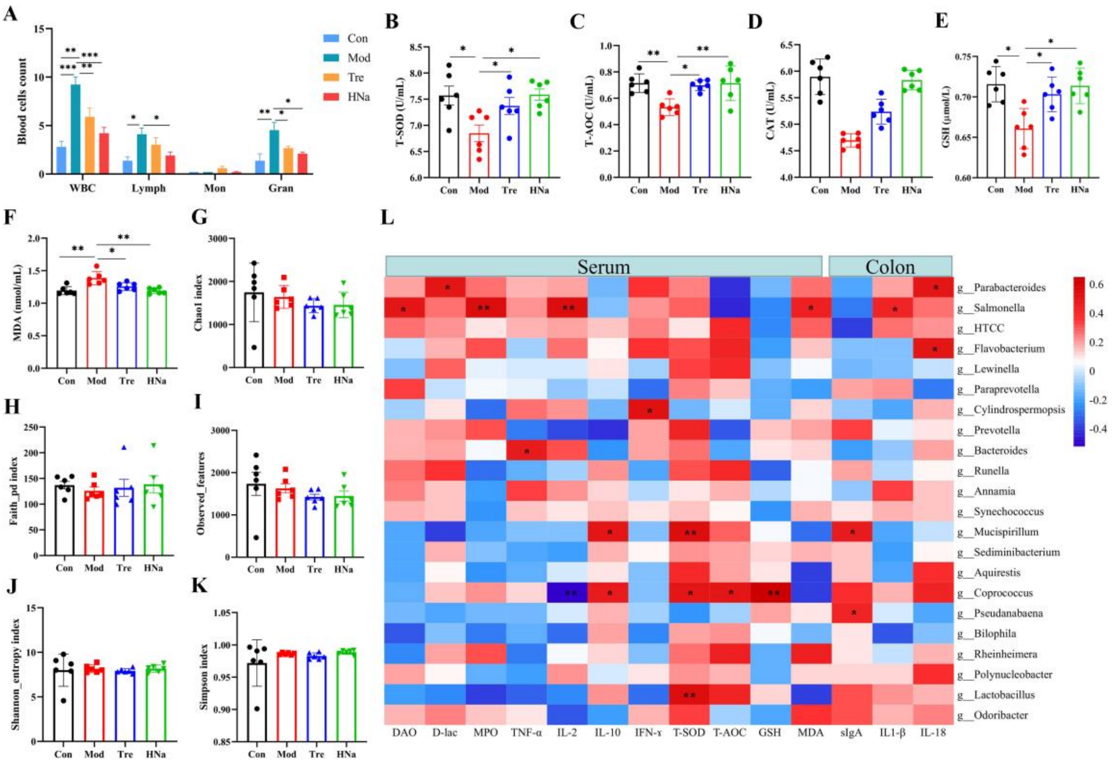

FIG S2 Administration of sodium humate (HNa) attenuated oxidative stress and regulated gut microbiota. (A) Blood parameters of white blood cells (WBC), lymphocyte (Lymph), monocyte (Mon), and granulocyte (Gran). The levels of serum total superoxide dismutase (T-SOD), total antioxidant capacity (T-AOC), catalase (CAT), glutathione (GSH), and malondialdehyde (MDA). (B-F). The alpha diversity index analysis of gut microbiota was represented by the Chao 1 (G), Faith-pd (H), Obaerved-features (I), Shannon (J), and Simpson (K). (L) Spearman correlation between gut microbiota and inflammatory or anti-oxidative parameters. The red color denotes a positive correlation, while blue color denotes a negative correlation. The intensity of the color is proportional to the strength of Spearman correlation. $^{*}P < 0.05$, $^{**}P < 0.01$. Statistical significance was determined using one-way ANOVA, followed by Tukey test. $^{*}P < 0.05$, $^{**}P < 0.01$, $^{***}P < 0.001$.

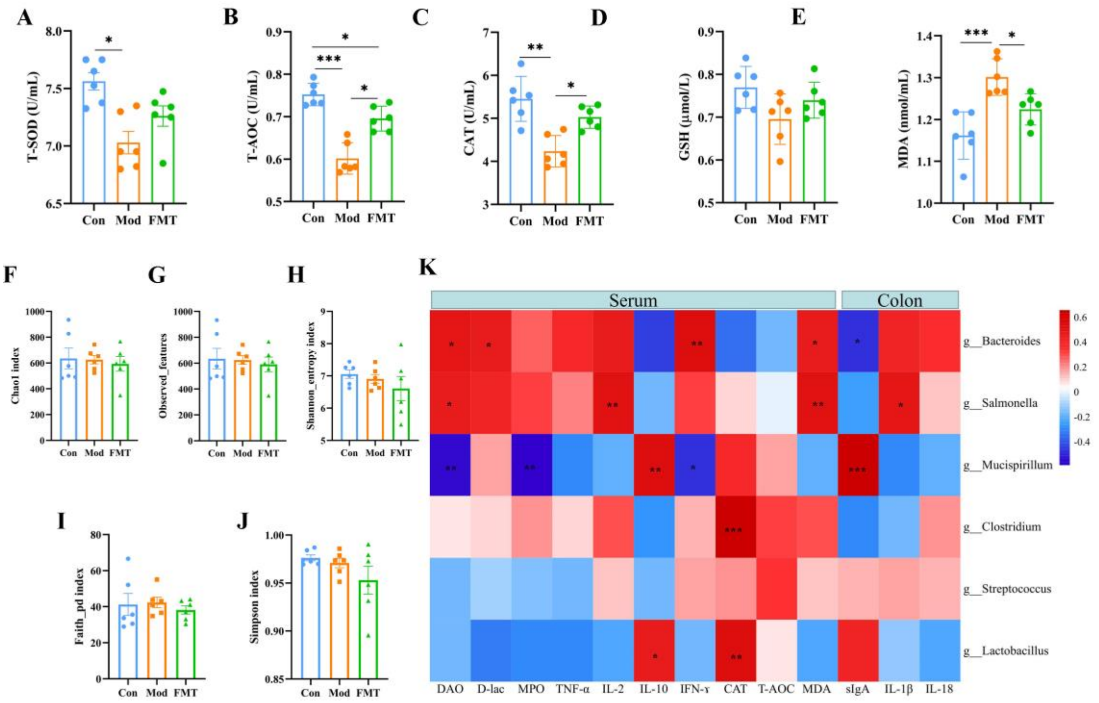

FIG S3 Sodium humate (HNa)-derived gut microbiota attenuated oxidative stress and regulated gut microbiota. (A-E) The levels of serum total superoxide dismutase (T-SOD), total antioxidant capacity (T-AOC), catalase (CAT), glutathione (GSH), and malondialdehyde (MDA). The alpha diversity index analysis of gut microbiota was represented by the Chao 1 (F), Obaerved-features (G), Shannon (H), Faith-pd (I), and Simpson (J). (K) Spearman correlation between gut microbiota and inflammatory or anti-oxidative parameters. The red color denotes a positive correlation, while blue color denotes a negative correlation. The intensity of the color is proportional to the strength of Spearman correlation. $^{*}P < 0.05$, $^{**}P < 0.01$. Statistical significance was determined using one-way ANOVA, followed by Tukey test. $^{*}P < 0.05$, $^{**}P < 0.01$, $^{***}P < 0.001$.

Supplementary Table 1. Primer sequences used for quantitative real-time PCR.

Genes	Sequence (5'→3')	Reference
flgG	F: CGCCGGACGATTGC<br>R: CCGGGCTGGAAAGCATT	Shi et al.,2019
motA	F: CGCGGCGAAAGTTATTTCAA<br>R: CCACGGACGCGGTATCC	Upadhyaya et al.,2013
sopB	F: GCGTCAATTTCATGGGCTAAC<br>R: GGCGGCGAACCCTATAAACT	Upadhyaya et al.,2013
invH	F: CCCTTCCTCCGTGAGCAAA<br>R: TGGCCAGTTGCTCTTTCTGA	Upadhyaya et al.,2013
sipA	F: CAGGGAACGGTGTGGAGGTA<br>R: AGACGTTTTTGGGTGTGATACGT	Upadhyaya et al.,2013
sipB	F: GCCACTGCTGAATCTGATCCA<br>R: CGAGGCGCTTGCTGATTT	Upadhyaya et al.,2013
hflK	F: AGCGCGGCGTTGTGA<br>R: TCAGACCTGGCTCTACCAGATG	Upadhyaya et al.,2013
lrP	F: TTAATGCCGCCGTGCAA<br>R: GCCGGAAACCAAATGACACT	Upadhyaya et al.,2013
ompR	F: TGTGCCGGATCTTCTTCCA<br>R: CTCCATCGACGTCCAGATCTC	Upadhyaya et al.,2013
tatA	F: AGTATTTGGCAGTTGTTGATTGTTG<br>R: ACCGATGGAACCGAGTTTTTT	Upadhyaya et al.,2013
16S	F: CCAGGGCTACACACGTGCTA<br>R: TCTCGCGAGGTCGCTTCT	Upadhyaya et al.,2013

F: Forward primer; R: Reverse primer.

Supplementary Table 2. Primer sequences used for quantitative real-time PCR.

Primer[1]	Sequence (5'→3')	Product size, bp	GeneBank accession No.
IL-2	F: GGCATTCGGGCTCCTTTCTT<br>R: TGGAGTGGTAGTCGATGCTAAG	132	NP_038633
Mucin-2	F: AGGGCTCGGAACTCCAGAAA<br>R: CCAGGGAATCGGTAGACATCG	106	AJ511872
TNF-α	F: CCGAGAGCGGAAGTGTGTG<br>R: TGTAACTGTGGTTTTGGTCTTCA	101	AF027131
IFN-γ	F: ATGAACGCTACACACTGCATC<br>R: CCATCCTTTTGCCAGTTCCTC	182	NM_008337
IL-10	F: GCTCTTACTGACTGGCATGAG<br>R: CGCAGCTCTAGGAGCATGTG	105	NM_010548
TLR4	F: ATGGCATGGCTTACACCACC<br>R: GAGGCCAATTTTGTCTCCACA	129	NM_021297
MyD88	F: TCATGTTCTCCATACCCTTGGT<br>R: AAACTGCGAGTGGGGTCAG	175	NM_010851
IKKα	F: ACAGCCAGGAGATGGTACG<br>R: CAGGGTGACTGAGTCGAGAC	297	NM_010546
IKB	F: AAGCACCCCTGGAAGAACC<br>R: CCTGCTCTGAAGGCAGATGTA	142	NM_178590
NF-κB	F: ATGGCAGACGATGATCCCTAC<br>R: TGTTGACAGTGGTATTTCTGGTG	111	NM_008689
NLRP3	F: ATTACCCGCCCGAGAAAGG<br>R: TCGCAGCAAAGATCCACACAG	141	NM_145827
ASC	F: CTTGTCAGGGGATGAACTCAAAA<br>R: GCCATACGACTCCAGATAGTAGC	154	NM_023258
Caspase-1	F: ACAAGGCACGGGACCTATG<br>R: TCCCAGTCAGTCCTGGAAATG	237	NM_009807
IL-18	F: GACTCTTGCGTCAACTTCAAGG<br>R: CAGGCTGTCTTTTGTCAACGA	169	NM_008360
Ki67	F: ATCATTGACCGCTCCTTTAGGT<br>R: GCTCGCCTTGATGGTTCCT	104	NM_0010811<br>17
Lysozyme	F: ATGGAATGGCTGGCTACTATGG<br>R: ACCAGTATCGGCTATTGATCTGA	214	NM_013590
PCNA	F: TTTGAGGCACGCCTGATCC<br>R: GGAGACGTGAGACGAGTCCAT	135	NM_011045
EGFR	F: GCCATCTGGGCCAAAGATACC<br>R: GTCTTCGCATGAATAGGCCAAT	101	NM_207655
β-actin	F: GGCTGTATTCCCCTCCATCG<br>R: CCAGTTGGTAACAATGCCATGT	154	NM_007393

[1]IL-2: Interleukin 2; TNF-α: Tumor necrosis factor α; IFN-γ: Interferon γ; IL-10: Interleukin 10; TLR4: Toll-like receptor 4; MyD88: Myeloid differentiation primary response gene 88; IKKα: Inhibitor of kappa B kinase α; IKB: Inhibitor of kappa B;

NF-κB: Nuclear factor kappa-B; NLRP3: NLR family pyrin domain containing 3; ASC: Apoptosis-associated speck-like protein containing a card; IL-18: Interleukin 18; PCNA: Proliferating cell nuclear antigen; EGFR: Epidermal growth factor receptor.
F: Forward primer; R: Reverse primer.
